# Mechanisms of Aflatoxin Detoxification: Adsorption and Inhibition Strategies

**DOI:** 10.3390/toxins18060244

**Published:** 2026-05-25

**Authors:** Yilin Tang, Lu Ding, Shujuan Sun, Mengmeng Mi, Minqi Shao, Yan Zhao, Mingxia Zhu, Yun Wang, Muhammad Zahoor Khan, Changfa Wang, Mengmeng Li

**Affiliations:** 1 School of Agriculture and Biology, Liaocheng Research Institute of Donkey High-Efficiency Breeding and Ecological Feeding, Liaocheng University, Liaocheng 252000, China; tangyilin0714@163.com (Y.T.); dinglu0708@163.com (L.D.); shaominqi2026@163.com (M.S.); yanzhao202604@163.com (Y.Z.); zhumingxia@lcu.edu.cn (M.Z.); zahoorkhan@lcu.edu.cn (M.Z.K.); wangcf1967@163.com (C.W.); 2 Liaocheng Academy of Agricultural Sciences, Liaocheng 252000, China; sunshujuan0528@163.com (S.S.); mimengmeng1997@163.com (M.M.); wangyun0206@163.com (Y.W.)

**Keywords:** aflatoxins, biological control, adsorption-type strategies, inhibition-type strategies, synergistic strategy, food safety

## Abstract

Aflatoxins (AFs), toxic secondary metabolites produced by *Aspergillus* species, represent a major threat to food safety and public health due to their pronounced hepatotoxic, carcinogenic, and mutagenic effects. With increasing global contamination risks driven by climate change and agricultural practices, the development of effective detoxification strategies has become a critical priority. This review provides a comprehensive and mechanistic overview of current aflatoxin (AF) decontamination approaches, focusing on two principal pathways: adsorption and inhibition strategies. Adsorption mechanisms involve the physicochemical sequestration of aflatoxins by inorganic materials, biological adsorbents, and engineered nanocomposites, thereby reducing toxin bioavailability. In contrast, inhibition strategies target fungal growth, toxin biosynthesis pathways, or promote enzymatic and microbial degradation of aflatoxins, offering more specific and potentially sustainable control. We critically analyze the underlying mechanisms, advantages, and limitations of each approach, including issues related to specificity, environmental stability, safety, and interactions with food matrices. Particular emphasis is placed on the toxicological implications of detoxification processes, including the reduction in aflatoxin-induced health risks and the safety of degradation products. Finally, this review highlights the importance of integrating adsorption and inhibition strategies to achieve synergistic decontamination and detoxification effects. Future perspectives on multifunctional materials, biological control systems, and intelligent monitoring technologies are discussed to advance the development of efficient, safe, and sustainable aflatoxin mitigation strategies.

## 1. Introduction

**Figure 1:**
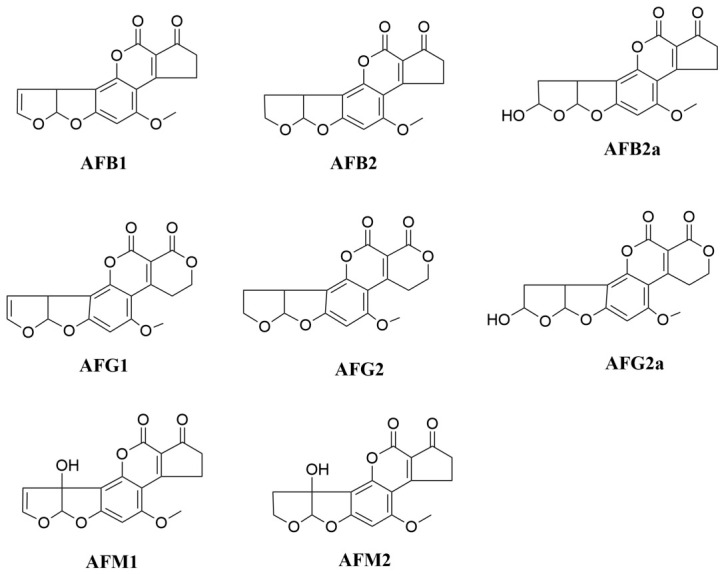
The chemical structures of different types of aflatoxins. From left to right and top to bottom: AFB1 (aflatoxin B1), AFB2 (aflatoxin B2), AFB2a (aflatoxin B2a), AFG1 (aflatoxin G1), AFG2 (aflatoxin G2), AFG2a (aflatoxin G2a), AFM1 (aflatoxin M1), and AFM2 (aflatoxin M2).

Aflatoxins (AFs) are a class of highly toxic secondary metabolites produced primarily by *Aspergillus flavus* (*A. flavus*) and *Aspergillus parasiticus*, alongside a few other *Aspergillus* strains, under specific environmental conditions [1]. These toxins possess complex chemical structures, with over 20 variants identified to date; among them, Aflatoxin B1 (AFB1), B2 (AFB2), G1 (AFG1), and G2 (AFG2) are the most prevalent (Figure 1). Upon ingestion by animals, AFB1 can be metabolically biotransformed into hydroxylated derivatives, such as Aflatoxin M1 (AFM1), which, despite exhibiting slightly lower toxicity than the parent compound, remains a significant hazard. AFs are ubiquitous in mold-susceptible agricultural commodities, such as peanuts, maize, rice, wheat, tree nuts, cottonseed, and so on. Due to their potent biological toxicity, AFs pose a severe and persistent threat to both food and feed safety [2].

Aflatoxins have been classified by the International Agency for Research on Cancer (IARC) as Group 1 carcinogens. Among them, AFB1 is generally regarded as the most toxic and best-characterized congener, owing to its pronounced hepatotoxicity and carcinogenic potential [3]. Its toxicological profile is multifaceted and severe. Primarily, AFB1 exhibits profound hepatotoxicity, capable of inducing acute liver injury, cirrhosis, and hepatocellular carcinoma through various mechanisms, representing one of the most critical threats to human and animal health [4,5]. Furthermore, AFB1 exerts detrimental effects on the reproductive system. In males, it induces apoptosis, inflammation, autophagy, cell cycle dysregulation, and oxidative stress, leading to reduced sperm production and increased abnormalities [6]. In females, it inhibits oocyte growth, reduces ovarian size and weight, and lowers 17β-estradiol concentrations. Crucially, the formation of covalent N7-guanine adducts interrupts deoxyribonucleic acid (DNA) replication, triggering chromosomal anomalies and fetal malformations [7].

AFs contamination not only incurs massive economic losses but also presents a serious challenge to public health, compromising livestock well-being [8] and human livelihoods [9]. Exacerbating this issue, climate change is projected to increase both the frequency and intensity of contamination events in the future [10].

More specifically, changes in temperature and humidity can reshape the ecological suitability and geographical distribution of aflatoxin-producing fungi. A nationwide analysis of 17,263 peanut AFB1 contamination records collected in China between 2009 and 2022 identified nighttime temperature, wind speed, and precipitation as key climatic drivers, with temperature variation accounting for 49.46% of the observed increase in contamination. Under a high-emission scenario, the same study projected that peanut AFB1 levels could reach 15.06 μg·kg^−1^ by 2100, while the quantity of peanuts exceeding the regulatory limit would increase from approximately 478,400 metric tons in 2022 to 1.16 million metric tons by 2100 [11]. In tropical feed systems, wet-season conditions associated with elevated temperature and humidity were linked to more severe contaminant profiles, including elevated aflatoxin prevalence. Furthermore, a recent systematic review concluded that heat, drought, and elevated CO_2_ may increase aflatoxin risk by 70–300% by 2050–2080 and promote the geographic expansion of aflatoxin-producing *Aspergillus* into newly vulnerable regions [10,12].

To address this multi-faceted challenge, contemporary research and practice generally follow two distinct trajectories. The first is the “adsorption-type” strategy, centered on adsorption or physicochemical sequestration, aimed at reducing the bioavailable toxin load during post-harvest storage or processing. The second is the “inhibition-type” strategy, which seeks to proactively mitigate toxin generation in the field or during storage by suppressing fungal growth or directly intervening in toxin biosynthetic pathways. These approaches differ significantly in their underlying principles and application scenarios. Clearly distinguishing the fundamental differences between these mechanisms, while exploring their respective advancements, limitations, and potential for synergy, is of paramount theoretical and practical significance for constructing a comprehensive AFs management system.

Inhibition-based strategies have been validated in studies involving small molecules, chemical inhibitors, and biotechnological interventions. For example, cycloleucine suppresses AF production by inhibiting N^6^-methyladenosine (m^6^A) methylation of messenger RNA (mRNA) [13]. In peanuts, the overexpression of antifungal plant defensins, including *Medicago sativa* defensin 1 (MsDef1) and *Medicago truncatula* defensin 4.2 (MtDef4.2), combined with host-induced gene silencing (HIGS) of *aflM* and *aflP*, significantly reduced fungal infection and toxin accumulation in field trials [14]. However, the genomic diversity of *A. flavus* and the variability of the AF biosynthetic gene cluster may limit the durability and broad applicability of single-target approaches [15,16]. Moreover, transgenic and HIGS-based strategies still face challenges related to field adaptability, regulatory approval, and public acceptance [11].

Conversely, adsorption-type strategies and detoxification methods mediated by processing or natural components offer an alternative operational pathway. Interventions that harness the phytochemical content and antioxidant activity of common culinary agents such as garlic, ginger, cardamom, and black cumin have demonstrated potential for decontaminating AFs in red pepper products as well as during sautéing. Similarly, thermal processing has been shown to effectively reduce AFs levels [17]. However, these findings also highlight critical issues, including a strong dependence on complex food matrices, the need to fully elucidate detoxification mechanisms, and the necessity for rigorous safety assessments of degradation products. Therefore, existing evidence supports a paradigm shift towards coupling multiple strategies. To advance the field, systematic mechanistic investigations at the intersection of molecular epidemiology and food matrix science are essential to achieve safe, persistent, and robust control of AFs risks in the face of genetically diverse pathogens and complex food systems.

## 2. Adsorption-Type Mechanisms: Reducing AFs Bioavailability via Binding

**Figure 2:**
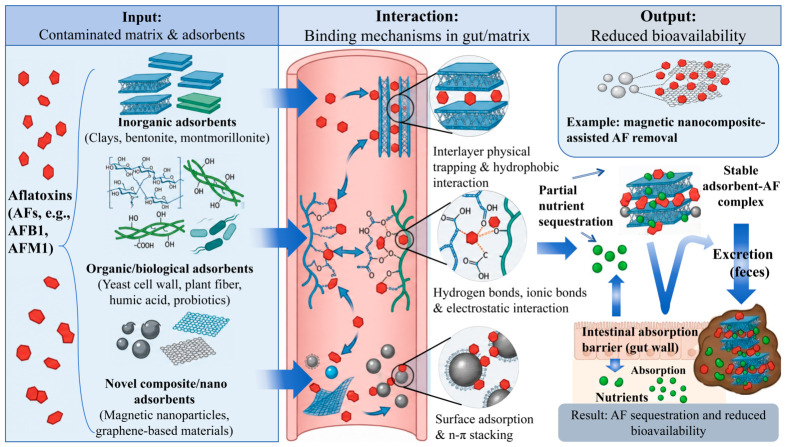
Schematic illustration of aflatoxin (AF) sequestration mechanisms by various adsorbents in the gastrointestinal tract. (**Left**) Aflatoxins (AFs, e.g., AFB1, AFM1) and three categories of adsorbents: inorganic adsorbents (clays, bentonite, montmorillonite), organic/biological adsorbents (yeast cell wall, plant fiber, humics, probiotics), and novel composite/nano adsorbents (magnetic nanoparticles, graphene-based materials). (**Middle**) Binding mechanisms in the gut/matrix including interlayer physical trapping and hydrophobic interaction, hydrogen bonds/ionic bonds/electrostatic interaction, and surface adsorption. (**Right**) Formation of stable adsorbent–AF complexes, including advanced magnetic nanocomposites, sequesters aflatoxins and reduces their intestinal absorption through the gut wall barrier, thereby lowering bioavailability and promoting excretion in feces; however, partial nutrient sequestration may also occur, while most nutrients remain available for normal absorption.

Adsorption strategies are categorized into three primary research domains: inorganic minerals, organic/biological adsorbents, and novel nanocomposite materials. Each class possesses distinct structural attributes and mechanistic advantages for AFs detoxification, yet each also faces specific limitations. This section systematically reviews the composition, structural characteristics, adsorption performance, and underlying mechanisms of these adsorbents (Figure 2). It critically evaluates the application of inorganic agents (e.g., montmorillonite, bentonite), organic/biological agents (e.g., yeast cell walls, phytogenic materials), and advanced magnetic nanocomposites in food and feed matrices. Furthermore, this review also examines the critical bottlenecks that impede practical applications, including low adsorption efficiency, limited specificity, poor regenerability, unresolved biosafety concerns, and inadequate cost-effectiveness. These analyses provide a robust theoretical foundation for the development of efficient, safe, and economically viable adsorbents.

### 2.1. Inorganic Adsorbents: Clay Minerals, Silicates, and Derivatives

Inorganic adsorbents, particularly clay minerals, carbon-based or porous minerals, silicates, and their derivatives, possess substantial potential for the sequestration of AFs, attributed to their distinct laminar structures and high specific surface areas.

Montmorillonite, a typical layered silicate clay mineral, has been extensively investigated as an aflatoxin adsorbent owing to its lamellar structure, high specific surface area, and exchangeable interlayer cations. Organic modification of its interlayer space can alter surface hydrophobicity, charge distribution, and the accessibility of toxin-binding sites, thereby improving its affinity for aflatoxins [18,19]. For example, chlorophyll-modified calcium montmorillonite (CMCH) exhibited a higher maximum adsorption capacity for AFB1 than unmodified calcium montmorillonite, with Qmax values of 0.43 and 0.34 mol/kg, respectively [20]. Its AFB1-binding performance was largely maintained under acidic and near-neutral pH conditions, suggesting that CMCH may retain its toxin-sequestering function across major gastrointestinal pH environments. Molecular modeling further indicated that hydrophobic interactions play an important role in AFB1 binding, while negative Gibbs free energy values (ΔG < 0) supported the thermodynamic favorability of the adsorption process [20].

These findings, however, should be interpreted with caution from the perspectives of safety and practical application. The stability of AFB1 binding at pH 2 and pH 6 [21] does not necessarily indicate that CMCH remains structurally intact throughout the entire gastrointestinal digestion process. Nor does it exclude the possibility that chlorophyll or chlorophyll-derived compounds may be released in the presence of digestive enzymes, bile salts, competing nutrients, and complex food or feed matrices [22]. This distinction is important because chlorophylls may undergo chemical transformation during digestion, yielding derivatives such as pheophytins and pheophorbides. In addition, although chlorophyll is a bio-based modifier, the chemical modification of montmorillonite should not be regarded as an inherently green process [18]. Therefore, before CMCH can be considered suitable for practical food or feed applications, future studies should directly quantify chlorophyll leaching, pigment transformation, AFB1 desorption, and post-digestion toxicity using simulated gastrointestinal digestion models [23].

Similarly, bentonite also demonstrates exceptional utility in diverse practical applications [24]. In PBS, HAFR series bentonites were tested at 0.5 and 1 g per 15 mL, and HAFR 3 achieved complete AFM1 adsorption after 12 h at the 1 g dose. In milk, 1 g of HAFR 3 was added to 30 mL of contaminated milk and reduced AFM1 from 100 ng/L to 1.5 ng/L after 12 h, corresponding to 98.5% adsorption [25]. Similarly, in hazelnut milk, bentonite effectively sequestered AFB1 and AFG1, thereby minimizing contamination loads and preventing economic losses in the nut industry [26].

In animal husbandry, a mycotoxin adsorbent (MAB) composed of bentonite and yeast cell wall was shown to alleviate AFB1-induced toxicity in laying hens consuming contaminated feed (0.2 mg/kg). MAB treatment restored performance metrics, egg quality, and organ health indices, while downregulating the overexpression of inflammatory and apoptotic markers (IFN-α, CASPASE-3/8) in uterine tissue [27]. In dairy cattle, daily supplementation (25 g/head) of commercial clay-based binders reduced milk AFM1 levels by 24.4% and 48.9% over 4 and 7 days, respectively, concurrent with improvements in milk composition [28]. In aquaculture, Georgian bentonite (“Askangel”) served as an effective natural adsorbent in trout feed; at inclusion rates of 0.15–0.2%, it achieved 83–90% AFB1 adsorption and significantly improved weight gain, survival rates, and feed conversion ratios [29].

Furthermore, other silicates such as silicoglycidol (ATX) have been shown to reduce the carry-over of AFM1 into milk and urine in dairy cows, without compromising lactation performance or hepatic biomarkers [30]. Comparative studies indicate that while mineral adsorbents like bentonite and clinoptilolite differ in binding capacity compared to organic β-glucans [31], they remain highly effective.

Although inorganic adsorbents have shown considerable potential for AF sequestration, their performance can vary substantially across different food, feed, and gastrointestinal environments. Several physicochemical factors contribute to this variability. Changes in pH may alter the surface charge and protonation state of both adsorbents and toxin molecules, thereby affecting electrostatic attraction, hydrogen bonding, ion exchange, and interlayer binding. Temperature can also influence adsorption by modifying molecular diffusion, mass transfer, and the thermodynamic behavior of toxin–adsorbent interactions. In addition, the initial toxin concentration determines the extent to which available binding sites become saturated; high removal efficiencies observed at low toxin levels may therefore decline when adsorption sites become limiting. Ionic strength and buffer composition further complicate the process, particularly for clay minerals, as exchangeable cations such as Ca^2+^, Mg^2+^, and Na^+^ can participate in or compete with toxin binding [32,33,34].

Beyond these environmental effects, non-specific adsorption remains a major practical concern. Inorganic adsorbents may bind essential nutrients together with toxins, making formulation optimization necessary to balance detoxification efficacy with nutritional integrity [35]. These constraints suggest that the practical value of AF adsorbents depends not only on intrinsic binding capacity, but also on material structure, surface chemistry, matrix compatibility, and the rational combination of complementary adsorbent classes. Accordingly, the advantages and limitations of representative adsorbent systems are summarized in need, followed by a discussion of combined adsorbent strategies (Table 1).

Different adsorbent categories have distinct structural features, binding mechanisms, and matrix compatibilities. However, a single material rarely meets all requirements for practical AF control, including high adsorption capacity, strong selectivity, matrix stability, and nutritional safety. In response to these limitations, the combined use of different adsorbents has become an important strategy for improving AF mitigation. For example, a composite adsorbent prepared from bentonite and yeast cell wall extracts was evaluated for its AFB1-binding capacity under in vitro conditions. The material retained an AFB1-binding efficiency of approximately 90% even at a low inclusion rate and high toxin concentration; when the adsorbent dose was increased and the toxin level was lower, the adsorption rate approached 99.6%. These findings suggest considerable formulation-optimization potential for mineral–biological composite systems [37].

Additional comparative evidence supports this view. When bentonite, yeast cell wall, and *Lactobacillus rhamnosus* cell wall were tested individually and in combination, the treated bentonite alone showed high AFB1 adsorption, whereas the combined system reached an AFB1 adsorption rate of approximately 94.50%. This result indicates that multi-component adsorbents may provide broader coverage in scenarios involving co-contamination with multiple mycotoxins [42]. Nevertheless, combining adsorbents does not necessarily produce an additive or synergistic effect. In an in vitro comparison of natural bentonite, acid-activated bentonite, yeast cell wall, and activated carbon, acid-activated bentonite bound more than 87%, and in some conditions more than 99%, of AFB1 at specific AFB1-to-binder ratios, whereas the performance of yeast cell wall and activated carbon varied with inclusion rate and experimental conditions [40].

These observations highlight the need to distinguish true synergistic adsorption from a simple increase in total adsorbent dosage. If different components interfere with one another through pore blocking, surface-charge neutralization, or competition for binding sites, the performance of a composite adsorbent may fall below theoretical expectations. Therefore, the design of composite adsorbents should be guided by mechanistic compatibility, optimized component ratios, and validation in realistic food, feed, or gastrointestinal matrices.

### 2.2. Organic and Biological Adsorbents: Yeast Cell Walls, Phytogenic Materials, and Humic Acids

Organic and biological adsorbents have garnered significant attention in the field of AFs control, driven by their natural origin, biocompatibility, and potential nutritional synergisms [43].

Yeast cell wall (YCW) preparations are among the most established biological adsorbents [44,45]. Research indicates that dietary supplementation with YCW significantly mitigates the hepatic deposition of AFB1 and its metabolite AFM1 in growing pigs, proving effective even at low contamination levels (20 µg/kg) [46]. Mechanistically, the sequestration capacity of YCW exceeds 92% in the in vitro studies and is attributed to its complex reticular structure, composed primarily of β-glucans and mannans. These components provide specific chemical sites that bind AFs through a combination of hydrogen bonding, ionic interactions, and hydrophobic forces, thereby preventing toxin absorption within the gastrointestinal tract [47].

Beyond yeast, phytogenic materials exhibit remarkable adsorption potential [48]. Alfalfa leaf meal, utilized as a natural adsorbent, has been shown to attenuate AFB1 toxicity by reducing its bioavailability [49,50,51]. In turkey poults fed AFB1-contaminated diets, the inclusion of alfalfa leaf meal not only improved growth performance (body weight and gain) and reduced feed intake but also significantly ameliorated AFB1-induced serum biochemical aberrations and hepatic histopathological damage [52]. Further mechanistic investigations reveal that alfalfa leaf meal functions by modulating the intestinal microbiota, specifically by promoting beneficial genera such as *Faecalibacterium* and *Coprococcus catus* while suppressing opportunistic pathogens like *Streptococcus lutetiensis*. This microbial modulation restores gut homeostasis, increases villus height, enhances intestinal barrier function, and reduces fluorescein isothiocyanate-dextran (FITC-d) permeability, thereby effectively counteracting the adverse effects of AFB1 [53].

Similarly, cereal derivatives such as corn, wheat, oat bran [54], and rice bran [55] demonstrate high AFB1 adsorption efficiency. Under specific pH and temperature conditions, corn bran achieved adsorption rates exceeding 78%, with binding sites predominantly localized in the pericarp fraction. Novel substrates like blackberry seed residues, rich in fiber and polyphenols, exhibited in vitro adsorption efficiencies ranging from 85.36% to 87.01% [56]. Corn by-products have also been explored for reducing AFB1 levels in both liquid and solid food matrices [57].

Humic acids (HAs) and their derivatives represent another critical class of organic adsorbents [58]. Characterized by abundant carboxyl and phenolic hydroxyl moieties, HA molecules sequester AFB1 via complexation, electrostatic interactions, and hydrogen bonding. In simulated poultry digestion models, natural humic acid derived from vermicompost and sodium-free humic acid (SFHA) achieved adsorption rates of 97.6% and 99.7%, respectively, significantly outperforming reference zeolite materials [59]. In vivo trials confirmed that 0.25% HA supplementation in turkey diets effectively alleviated growth inhibition, hepatic injury, and serum biochemical anomalies caused by AFB1 exposure [60].

Furthermore, probiotics and their metabolites act as potent sequestering agents. Lactic acid bacteria (e.g., *L. plantarum*, *L. casei*, *L. paracasei*) bind AFs molecules via cell wall components to form stable toxin–microbe complexes [61]. Notably, *L. paracasei* achieved removal rates of 71.35% in phosphate-buffered saline and 68.53% in milk [62]. Peptidoglycans isolated from *Limosilactobacillus reuteri* demonstrated in vitro adsorption efficiencies of 64.3–75.9% and significantly improved growth retardation, immunosuppression, and liver pathology in AFB1-exposed broilers [63]. Similar efficacy has been reported for *Bifidobacterium*, *Saccharomyces cerevisiae* [64], and probiotic consortia [65]. Chitosan, a natural biopolymer, is also utilized for toxin removal due to its unique adsorption properties [66].

Although organic and biological adsorbents have advantages in terms of biocompatibility, multifunctionality, and potential compatibility with food or feed systems, their practical performance remains highly dependent on both material properties and matrix conditions. Yeast cell wall-derived β-glucans provide a representative example. In an in vitro comparison of bentonite, clinoptilolite, and yeast cell wall β-glucans, the yeast-derived additive adsorbed 79.43–89.32% of AFB1 at pH 3 and 87.56–93.50% at pH 6. However, its calculated maximum adsorption capacity was lower than that of some mineral binders, with values of 3.4 mg/g at pH 3 and 2.3 mg/g at pH 6 [35]. These findings indicate that biological adsorbents can be effective, but their adsorption capacity may vary according to pH, toxin concentration, product composition, and the structural accessibility of binding sites.

Similar variability has been observed for lactic acid bacteria. A recent study reported that different LAB strains reduced AFB1 levels by 16–71%, depending on strain type, cell viability, and pH. Non-viable cells generally exhibited stronger binding capacity than viable cells, whereas biotransformation accounted for only 0–18% of AFB1 reduction [67]. Microbial adsorbents therefore should not be treated as a uniform category. Before practical application, strain selection, processing history, cell viability, and desorption stability need to be carefully evaluated.

Stability and specificity are also important limitations. Yeast cell wall products are attractive because their β-glucan-rich polysaccharide network can resist digestion and help maintain binding capacity during gastrointestinal transit. Indeed, yeast cell wall-based mycotoxin adsorbents have already been incorporated into animal feed as part of integrated mycotoxin management strategies [68]. Nevertheless, their efficacy varies among mycotoxins and formulations, and some products may perform better against relatively hydrophobic toxins than against others. Plant-derived adsorbents and agricultural by-products are more readily available and potentially cost-effective, but their performance is strongly influenced by botanical source, particle size, processing method, adsorption time, temperature, pH, and matrix composition. For instance, corn cob powder achieved up to 98% AFB1 adsorption under optimized conditions and reduced AFB1 levels in both liquid and solid food matrices; however, its adsorption efficiency was significantly affected by adsorbent dose, particle size, contact time, temperature, and pH [57].

### 2.3. Novel Composite and Nanomaterials

The limitations of traditional adsorbents regarding efficiency, selectivity, and recyclability have propelled the development of advanced composite and nanoscale adsorbents, such as carbon-based nanoparticles (CBNs) [69] and magnetic nanoparticles (MNPs) [70,71], aimed at achieving superior and more sustainable AFs removal.

Magnetic nanomaterials, distinguished by their unique physicochemical properties and facile separation capabilities, have garnered significant attention. For instance, magnetic reduced graphene oxide (MrGO) nanocomposites integrate magnetic nanoparticles with high-surface-area graphene oxide derivatives, thereby providing abundant binding sites that significantly enhance adsorption efficiency for AFB1 [72]. A salient advantage of these materials lies in their magnetic responsiveness: upon saturation, separation can be rapidly achieved via an external magnetic field. This feature drastically simplifies the complexity associated with traditional filtration or centrifugation, effectively reducing operational costs and mitigating potential environmental risks, thus underscoring their immense potential for practical application.

Reusability is closely linked to both cost and environmental performance. A dopamine-grafted biomass chitosan–iron–cobalt spinel oxide nanocomposite retained AFB1 removal efficiencies of 81.30% in water and 72.20% in edible oil after six reuse cycles, with HCl showing better desorption performance than NaOH [73]. These results demonstrate the feasibility of regenerating magnetic nanocomposites, but they also indicate that repeated use commonly requires acid/base- or solvent-assisted desorption, followed by washing and material recovery. Such procedures may increase operational costs, generate wastewater treatment demands, and raise additional food safety validation concerns. Moreover, even when an adsorbent remains reusable over multiple cycles, its adsorption capacity, desorption efficiency, and potential release of residual nanoparticles should be monitored throughout repeated use.

Furthermore, the engineering of heterogeneous composite materials to leverage synergistic effects among components represents a current research hotspot. Polymer-based composites [74], such as lignin–chitosan–iron nanocomposites, have been developed to provide robust solutions for AFs removal in food and feed systems [75]. These composites typically capitalize on the rich functional groups (e.g., phenolic hydroxyls, amino groups) inherent in natural polymers like lignin and chitosan, which facilitate toxin binding through hydrogen bonding, n-π stacking, and electrostatic interactions. Concurrently, the incorporation of iron nanoparticles not only enhances structural stability and mechanical strength but may also introduce additional redox activity or adsorption sites, further amplifying adsorption capacity and selectivity.

This design philosophy of multi-component synergy enables composite materials to address some intrinsic deficiencies of single-component systems and can improve overall adsorption performance under laboratory conditions. Nevertheless, before these materials are proposed as economically viable technologies for AFs decontamination in real-world food and feed matrices [76], their cost-effectiveness, scalability, regeneration stability, matrix compatibility, and long-term safety should be systematically validated.

Despite these advantages, the practical deployment of heterogeneous composite and nanomaterial-based adsorbents should be assessed beyond adsorption efficiency alone, with due consideration of production cost, scalability, and reusability. Polymer–metal or magnetic composites are generally prepared through a series of sequential processing steps, including polymer extraction or purification, metal precursor loading, crosslinking, surface functionalization, repeated washing, drying, and, in some cases, thermal treatment. These procedures should not be regarded merely as routine laboratory operations, as they are closely associated with the production cost, scale-up feasibility, and environmental footprint of the final material. For instance, polymer extraction or purification is often required to obtain chitosan, lignin, cellulose, or other biopolymers with sufficient purity and stable functional groups; however, this process may involve considerable consumption of chemical reagents, water, and energy. The incorporation of metal species or magnetic nanoparticles further requires additional metal salts or precursors, as well as carefully controlled reaction conditions to ensure a uniform distribution of active sites [75,76]. Crosslinking and surface functionalization are commonly employed to enhance adsorption capacity, structural stability, and selectivity, but they may introduce extra reagents, catalysts, pH adjustment procedures, prolonged reaction times, and post-treatment requirements [71]. Subsequent washing and drying are essential for removing unreacted chemicals, residual ions, and loosely attached nanoparticles, yet these operations may generate wastewater and increase energy demand [73]. In some cases, thermal treatment is also applied to improve structural stability or magnetic performance; however, this step adds further energy input and may restrict the use of heat-sensitive biopolymers. Although these modification strategies can improve adsorption performance under laboratory conditions, these procedures may substantially increase reagent consumption, energy input, wastewater generation, and overall manufacturing costs, particularly when high-purity polymers, metal precursors, or precisely controlled nanostructures are required [77]. Therefore, their potential benefits need to be carefully balanced against preparation complexity, economic feasibility, secondary waste generation, and environmental sustainability when considering large-scale applications in food or feed systems.

Scalability represents another critical challenge. Synthetic protocols that perform well at the laboratory scale do not necessarily ensure batch-to-batch reproducibility, uniform particle size distribution, stable surface chemistry, or consistent adsorption performance under industrial food and feed processing conditions. Reusability may help offset production costs, especially for magnetic or membrane-supported composites that can be readily recovered after treatment. However, repeated use usually requires solvent washing, acid/base desorption, or other regeneration procedures. Over successive cycles, these treatments may reduce adsorption capacity and create additional requirements for waste management and food-safety validation [78].

Accordingly, future studies should move beyond reporting removal efficiency and adsorption capacity alone. More comprehensive evaluation metrics are needed, including synthesis yield, adsorbent cost per unit mass, treatment cost per unit matrix, regeneration efficiency over multiple cycles, potential nanoparticle migration risks, and life-cycle environmental impacts.

### 2.4. Limitations of Adsorption-Type Mechanisms

While adsorption-based AFs sequestration and decontamination agents have demonstrated promising efficacy in both in vitro and in vivo models, their transition to widespread practical application is impeded by several substantial limitations.

Foremost among these challenges are adsorption efficiency and specificity. The binding capacity of many adsorbents is highly sensitive to environmental parameters, including pH, temperature, ionic composition, and coexisting macromolecules. For example, recent studies on bentonite have shown that AFB1 adsorption differs between acidic and neutral buffer systems. Adsorption maxima are generally higher at pH 3 than at pH 7, and buffer composition can significantly influence the adsorption process [32]. Similar matrix-dependent effects have been observed in simulated gastric fluid, where the adsorption capacity of Ca-montmorillonite decreased from 0.52 mol/kg in water to 0.32 mol/kg. This reduction was mainly attributed to the ability of pepsin and other components to enter the interlayer space and compete with AFB1 for adsorption sites [79]. The type of exchangeable cation is another important determinant of adsorption performance. Smectites saturated with divalent cations, particularly those with smaller hydrated radii, showed stronger AFB1 adsorption than Na-smectite, indicating that cation valence, hydrated radius, and layer-charge density jointly regulate toxin affinity [80]. Temperature may further modify adsorption behavior by affecting molecular mobility, diffusion rates, and adsorbent structure. In a biological adsorption model, AFB1 uptake increased from 66 ± 3% at 22 °C to 85 ± 3% at 50 °C, suggesting an endothermic adsorption process [81]. For clay minerals, thermal treatment of bentonite has also been shown to alter AFB1 adsorption capacity; excessive heating can reduce adsorption performance because of structural changes in the clay matrix [82]. This often results in a performance that falls significantly short of theoretical maxima when applied in complex food or feed matrices.

Conventional non-specific adsorbents, such as montmorillonite and zeolite, may also bind essential nutrients and thereby reduce their gastrointestinal bioavailability. As a result, they can non-specifically sequester vitamins, minerals [83], proteins [84], and other nutrients, potentially compromising the nutritional integrity of feed and adversely affecting animal health and production performance. In an in vitro post-ruminal digestion model, six mycotoxin binders showed varying capacities to adsorb amino acids and water-soluble vitamins. When incubated separately, the average adsorption rate of amino acids was 44.3%, whereas that of water-soluble vitamins ranged from 34.1% to 45.1%; notably, montmorillonite adsorbed as much as 90.5–97.0% of vitamin B1 [85]. In a poultry gastrointestinal model, aluminosilicate clays were also found to sequester essential trace minerals, including Fe, Mn, Zn, and Se. One beidellite–Ca-montmorillonite clay adsorbed 95.5% of Fe, 94.5% of Se, 72.0% of Zn, and 52.5% of Mn [86]. Proteinaceous components may further interfere with toxin binding, as pepsin can enter montmorillonite interlayers and compete with AFB1 for adsorption sites [79]. Together, these findings indicate that non-specific adsorption may impair nutrient bioavailability, particularly when binders are used at high doses or over extended periods.

Regeneration remains another practical challenge. Most feed binders are designed to sequester toxins during gastrointestinal passage and then be excreted in the feces, rather than to be recovered and reused. Although some engineered adsorbents can be regenerated, the procedures are often technically demanding. For example, magnetic chitosan–iron–cobalt composites can be recovered magnetically and subsequently desorbed using solvents or acidic/alkaline media [73], while covalent organic framework fiber membranes can be regenerated through alkaline washing [87]. Such processes, however, introduce additional requirements for separation, solvent consumption, pH adjustment, waste handling, and cost control [78]. Therefore, the feasibility of adsorbent regeneration should be evaluated not only in terms of adsorption performance, but also with respect to food/feed safety, environmental impact, and economic practicality.

Safety concerns constitute a non-negligible barrier to application. Certain adsorbents may inherently contain hazardous impurities or generate secondary contaminants during use. For instance, natural clay minerals pose potential risks of contamination with dioxins or heavy metals such as lead and cadmium. Furthermore, the physicochemical properties of adsorbents, particularly particle size and morphology, may adversely impact gastrointestinal health in animals.

Finally, cost-effectiveness remains a decisive factor limiting widespread adoption. High-performance, safe adsorbents often command a premium price, presenting a significant economic barrier for large-scale feed production and food processing. In many scenarios, the cumulative cost of preventive measures or rigorous raw material screening may prove lower than the long-term administration of high-end adsorbents, rendering the latter economically uncompetitive in certain contexts. Consequently, the development of adsorbents that simultaneously offer high efficiency, superior selectivity, robust regenerability, biosafety, and cost-effectiveness remains an urgent and unresolved challenge in the field.

## 3. Inhibition and Degradation Strategies: Controlling AF Formation and Pre-Formed Toxins

**Figure 3:**
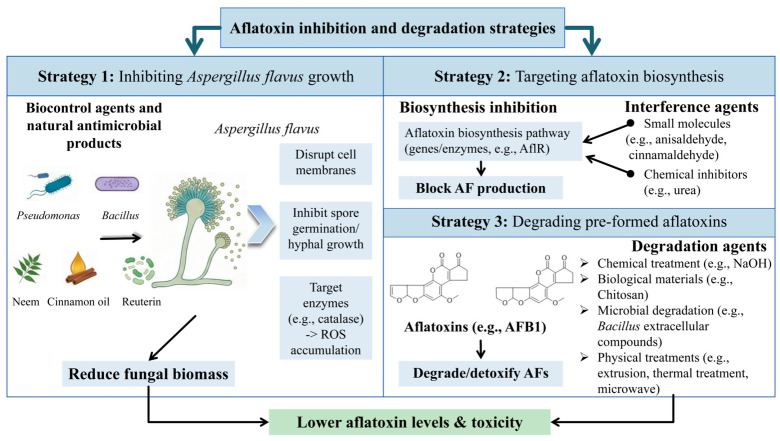
Overview of aflatoxin inhibition and degradation strategies. Strategy 1 (**Left**) Inhibiting *Aspergillus flavus* growth through natural antagonists/biocontrol bacteria, such as *Pseudomonas* spp. and *Bacillus* spp., together with naturally derived antimicrobial products, including neem oil, cinnamon oil, and the fermentation-derived metabolite reuterin. These agents disrupt fungal cell membranes, inhibit spore germination/hyphal growth, and target key enzymes (e.g., catalase) and reactive oxygen species (ROS) accumulation, resulting in reduced fungal biomass. Strategy 2 (**Upper right**) Inhibition of aflatoxin biosynthesis by targeting pathway-associated genes and regulatory enzymes, represented here by AflR. Small molecules, including anisaldehyde and cinnamaldehyde, as well as chemical inhibitors such as urea, can interfere with aflatoxin biosynthetic regulation and consequently suppress AF production. Strategy 3 (**Lower right**) Degradation or detoxification of preformed aflatoxins, exemplified by AFB1, using chemical treatments, biological materials, microbial extracellular compounds, and physical processing methods, including thermal treatment, ultrasound, and microwave treatment. By acting on fungal proliferation, toxin biosynthesis, and existing toxin residues, these strategies collectively contribute to lower aflatoxin levels and reduced toxicity.

In contrast to terminal adsorption-based detoxification strategies, inhibition-based strategies—operating at the frontend (pre-harvest) and midstream stages—offer a more proactive approach by blocking fungal growth or targeting toxin biosynthesis and degradation. By intervening at the source, these methods have emerged as a core research direction for controlling AFs contamination. Currently, inhibition mechanisms fall into three primary categories: (1) disrupting the biological basis of toxin production by interfering with the growth and reproduction of *Aspergillus* via natural antimicrobials, microbial metabolites, and plant extracts; (2) achieving precision control at the molecular level by targeting key pathways in AFs biosynthesis; (3) employing chemical, biological and physical methods to degrade pre-formed toxins. (Figure 3). The following sections systematically review the principles, progress, and efficacy of these strategies, with a specific emphasis on the antifungal mechanisms of natural agents, key targets for biosynthetic inhibition, and degradation technologies. Furthermore, we provide a critical analysis of the limitations impeding the large-scale application of inhibition strategies—namely specificity, stability, and engineering challenges—to inform the development of high-efficiency, green, and cost-effective AFs control technologies.

### 3.1. Strategies Inhibiting Aspergillus Growth

Suppressing the proliferation of *A. flavus* constitutes a pivotal upstream strategy for controlling AF contamination. By effectively intervening in fungal growth and reproduction, this approach disrupts the biological foundation of toxin biosynthesis.

Current research has extensively explored natural antimicrobials, including microbial metabolites and plant-derived extracts, for this purpose. For instance, the crude extract (PHE) from the endophytic bacterium *Pseudomonas* sp. *HP-1*, isolated from *Peganum harmala* L., exhibits broad-spectrum antifungal activity. Its primary active compound, *1-phenazinecarboxylic acid*, functions by compromising cell membrane integrity to inhibit mycelial growth [88]. Similarly, culture filtrates from antagonistic strains *Burkholderia plantarii M1-8* and *Burkholderia glumae M6-4* have been confirmed to significantly suppress *A. flavus*. Notably, these filtrates maintain robust stability against *Proteinase K*, intense light, varying pH levels, and temperatures up to 70 °C [89], underscoring their potential application in complex environmental conditions.

Fermentation-derived products also display potent inhibitory properties [90,91]. Synergistic solid-state fermentation using *Weizmannia coagulans* and *Bacillus subtilis* not only enhanced nutritional value but also substantially reduced AFB1 content in peanut meal, providing indirect evidence of suppressed fungal growth or toxin production [92]. Furthermore, cell-free supernatants (CFS) from *Bacillus velezensis 906* induced severe morphological deformations and membrane disruption in *A. flavus*, leading to cell death and inhibition of spore germination, as confirmed by scanning electron and fluorescence microscopy [93]. At the molecular level, reuterin, a broad-spectrum antimicrobial from *Limosilactobacillus reuteri*, completely inhibited conidial germination at 6 mM. Molecular docking studies suggest that reuterin competitively binds to the active sites of key enzymes such as catalase, leading to lethal intracellular reactive oxygen species (ROS) accumulation [94]. This targeted enzymatic inhibition offers a precision pathway for novel antifungal development.

Plant-derived extracts present another promising frontier. Neem and lemongrass leaf powders have been shown to effectively suppress sporulation and toxin production; specifically, 5% (*w*/*w*) neem leaf powder inhibited *A. flavus* sporulation by 76.15% after 30 days of maize storage [95]. Cinnamon essential oil (CEO) demonstrated significant efficacy with a minimum inhibitory concentration (MIC) of 0.78 g/L. Its mechanism involves disrupting membrane permeability, inducing the leakage of intracellular contents, promoting malondialdehyde (MDA) accumulation, and reducing superoxide dismutase (SOD) activity [96]. These findings highlight the potential of essential oils to act via multi-target synergistic mechanisms [97]. While culinary spices such as garlic, ginger, cardamom, and black cumin are primarily noted for their AFB1 detoxification potential, it is plausible they also exert indirect control by inhibiting fungal proliferation [17]. Moreover, specific volatile compounds like 2-ethylhexanol have been confirmed to inhibit AFB1 production by downregulating genes related to ergosterol synthesis and global regulators [98], while the inhibitory mechanisms of 1-octene-3-ol are currently under investigation [99,100].

In addition to microbial and plant-derived inhibitors, processing-mediated decontamination can serve as an important post-harvest strategy for controlling AF contamination. Once toxins have already accumulated in raw materials, suppressing the growth of *A. flavus* alone is insufficient to eliminate the associated risk. In such cases, processing technologies such as extrusion, microwave treatment, ozonation, advanced oxidation, cold plasma, and pulsed light may be applied to further reduce residual toxin levels.

Extrusion processing has been shown to markedly decrease the apparent concentrations of AFB1, AFB2, AFG1, and AFG2 in corn-based matrices. However, part of this reduction may result from the binding of toxins to food macromolecules or the formation of modified toxin forms, rather than complete degradation. Therefore, in vitro digestion models and bioaccessibility assessments are needed to determine the actual detoxification efficacy [101]. Water-assisted microwave treatment and UV/H_2_O_2_-based advanced oxidation mainly promote AFB1 degradation through thermal effects, radical-mediated oxidation, and structural disruption, and both approaches have shown potential in corn or peanut matrices [102,103,104].

Nonthermal or mild-thermal technologies, including ozone treatment, cold plasma, and pulsed light, can also reduce AFB1 levels through reactive oxygen/nitrogen species, oxidative cleavage, or photochemical reactions. Notably, pulsed light may simultaneously inhibit *A. flavus* growth and suppress AF production [105,106,107]. Nevertheless, processing-mediated decontamination should not be evaluated solely on the basis of toxin reduction rates. Its practical application also requires careful assessment of nutritional and sensory quality, the toxicity of degradation products, scalability, and regulatory acceptance. Thus, food-processing decontamination may function as a downstream complement to fungal-growth inhibition, particularly in complex food matrices where toxin accumulation has already occurred.

Collectively, these studies elucidate a multidimensional strategy for suppressing *A. flavus* growth, providing a theoretical basis and practical direction for developing safe and efficient antifungal agents. Thus, future AF control strategies should integrate fungal-growth inhibition with post-harvest decontamination to achieve more reliable and adaptable contamination management.

### 3.2. Targeting AFs Biosynthesis and Degradation

Targeting AFs biosynthesis and the degradation of pre-formed toxins represents an important route for controlling contamination risk. Unlike strategies that mainly rely on terminal adsorption, these approaches act primarily at the molecular level. They can suppress AF formation by interfering with the fungal toxin biosynthetic machinery or reduce residual toxin levels and potential toxicity by degrading already-formed toxin molecules through microbial, enzymatic, chemical, or physical catalytic processes. In *A. flavus*, aflatoxin biosynthesis is governed by a clustered set of structural and regulatory genes, among which *aflR* and aflS are central regulatory components. *aflR* encodes AflR, a pathway-specific Zn(II)_2_Cys_6_-type transcription factor that recognizes and binds promoter regions of aflatoxin biosynthetic genes, thereby activating the transcription of downstream structural genes. *aflS*, located adjacent to *aflR*, is generally considered a co-regulatory factor of AflR. It modulates AflR activity and enhances AF production, although AflS itself does not function as an independent DNA-binding transcription factor [108]. Previous studies have shown that *aflR* expression is closely associated with AF accumulation, and that environmental conditions and nutritional factors can alter toxin biosynthesis by affecting AflR-mediated regulation [109]. In addition, the role of AflR may extend beyond the transcriptional activation of structural genes within the aflatoxin biosynthetic cluster, as it is also implicated in the growth, development, and broader physiological regulation of *A. flavus* [110]. Biochemical evidence further indicates that AflR and AflS functionally interact, and that this interaction can influence the DNA-binding affinity, stoichiometry, and binding kinetics of AflR.

Based on the above regulatory framework, studies on the inhibition of aflatoxin biosynthesis have mainly focused on two aspects: disrupting fungal metabolic pathways [111,112] and modulating the expression of key genes. AflR provides a representative example, given its important transcriptional regulatory role in the AF biosynthetic gene cluster. Researchers have attempted to interfere with this pathway using engineered peptides fused with the binuclear zinc-finger motif of AflR. These engineered peptides can bind to specific DNA recognition sites of transcriptional regulators, thereby suppressing the expression of key genes involved in toxin production and enhancing host resistance against toxigenic fungal infection [113].

Furthermore, various small-molecule compounds demonstrate significant inhibitory efficacy. Anisaldehyde and cinnamaldehyde exhibit synergistic antifungal activity against *A. flavus* growth and toxin accumulation [114], while methyl 2-methylbutyrate impedes AFs genesis through distinct mechanistic pathways [115]. Rhein, a natural anthraquinone, effectively arrests fungal development and AFs biosynthesis by disrupting cellular energy supply [116]. Among chemical inhibitors, urea [117], particularly in nano-formulations, shows immense potential. Standard urea at 3% achieved 100% growth inhibition and a 78.74% reduction in AFB1 yield, whereas nano-urea at 2% achieved total growth inhibition and an 83.36% reduction in AFB1 [118], underscoring that carrier optimization can significantly enhance inhibitory efficiency.

Conversely, strategies focused on the degradation of pre-formed AFs are equally critical. Chemical treatment is one effective approach; for example, treating low-grade maize with 5% (*w*/*v*) NaOH at 50 °C reduced AFB1 content to 4.25 µg/kg, achieving a 60.35% degradation rate [119]. Alkaline treatment is associated with opening of the lactone ring in the aflatoxin molecule, which can reduce its fluorescence and toxicity. Beyond chemicals, biological materials show broad prospects. Chitosan derived from edible mushroom residues completely inhibited *A. flavus* growth at 2% concentration and fully suppressed AFB1 production at 1.5% [120]. This effect is likely related to the cationic nature of chitosan, which can interact with negatively charged fungal cell surfaces, disturb membrane integrity, and impair cellular growth, thereby indirectly reducing the metabolic capacity required for AFB1 biosynthesis. Nano-encapsulation offers a pathway for essential oils; *Cymbopogon khasiana* × *Cymbopogon pendulus* essential oil (CKP-25-EO) encapsulated in chitosan nano-emulsion not only enhanced antifungal and anti-AFB1 activities but also interfered with ergosterol and methylglyoxal biosynthesis, presenting a green alternative for food preservation [121]. Additionally, microorganisms and their secretions possess potent degrading capabilities, damaging fungal cell wall and protein structures, thereby limiting fungal proliferation. Extracellular compounds from *Bacillus siamensis 3BS12-4* exhibited a 43.10% inhibition rate against *A. flavus* and achieved a remarkable 96.11% degradation of AFB1 [122].

Innovative composite materials have also been developed for targeted detoxification in liquid matrices. A novel polyvinylidene fluoride (PVDF) composite membrane incorporating Fe/Co-based metal–organic frameworks (Fe/Co-MIL-88B(NH_2_)) and CaO_2_ demonstrated higher degradation efficiency in the reported system. Leveraging its hierarchical pore structure and peroxidase-mimic catalytic activity, this system achieved >95% degradation of AFB1 and AFM1 within 60 min, with excellent reusability [123]. These studies highlight the dual importance of targeting biosynthetic pathways and degrading pre-formed toxins, while simultaneously emphasizing the limitations of isolated strategies in complex environments.

### 3.3. Limitations of Inhibition-Type Mechanisms

While inhibition-type mechanisms demonstrate immense potential in interrupting AFs biosynthesis, their translation into practical applications is currently impeded by a multifaceted array of challenges. The issue of specificity represents a fundamental constraint in current inhibition strategies. Many identified inhibitors exhibit narrow-spectrum activity, effective only against specific AFs congeners (e.g., AFB1, AFG1) or particular fungal strains [124,125]. In real-world scenarios characterized by co-contamination with multiple mycotoxins or the co-existence of diverse toxigenic strains, this insufficient broad-spectrum efficacy constitutes a major limitation, failing to provide comprehensive protection against the complex microbial ecology of food systems. Beyond specificity concerns, processing efficiency poses substantial hurdles for large-scale implementation. Achieving homogenous distribution of inhibitors within complex food or feed matrices, while maintaining effective concentrations throughout extended storage and processing periods to sustainably suppress fungal proliferation and toxin genesis, represents a formidable engineering challenge [126,127]. Inadequate application efficiency or uneven distribution not only compromises control outcomes but may also exacerbate food safety risks by creating pockets of uncontrolled fungal growth. Compounding these challenges, the environmental stability of inhibitors emerges as a critical determinant of their widespread utility. Under fluctuating conditions, including thermal stress during processing, humidity variations during storage, or pH shifts, many bioactive compounds are prone to rapid degradation or inactivation. This instability substantially attenuates their inhibitory potency, thereby restricting their applicability across diverse and harsh food and feed production environments [128,129,130]. Equally important are concerns regarding residues, safety, and matrix interactions, which cannot be overlooked in the development pathway toward commercial viability. Any additive intended for food or feed requires rigorous toxicological assessments to ensure that neither the parent compound nor its metabolites pose health risks to consumers or livestock [131,132,133]. Moreover, as consumer demand for high sensory quality intensifies, any inhibitor-induced adverse alterations in organoleptic properties (flavor, color, texture), or interference with the bioavailability of key nutrients (e.g., proteins, vitamins), would severely hinder their commercial adoption and regulatory approval [134].

## 4. Mechanism Synergy: Challenges and Future Directions

While adsorption-based and inhibition-based mechanisms each demonstrate distinct potential for controlling AFs, the application of any single mechanism is often constrained by inherent limitations. Consequently, there is a paradigm shift towards comprehensive solutions rooted in mechanism synergy (Table 2).

Regarding adsorption-type mechanisms, while diverse adsorbents demonstrate efficacy in binding AFs, their practical application is frequently compromised by issues of non-specific sequestration. This lack of selectivity can lead to the unintended removal of essential nutrients from feed or food matrices, thereby impairing their bioavailability. For instance, a study involving broilers fed diets contaminated with tolerance levels of AFB1 revealed that the co-administration of an adsorbent (Toxfin) and an antioxidant (Vitamin C) significantly enhanced the digestibility of dry matter, organic matter, crude protein, crude fiber, and nitrogen-free extract, as well as the assimilation of nitrogen and phosphorus [169]. These findings indirectly suggest that adsorbent monotherapy, in the absence of antioxidant support, may interfere with nutrient absorption, underscoring the potential drawbacks associated with non-specific binding. Furthermore, the adsorption efficiency for AFs is susceptible to environmental variables such as pH, temperature, and co-existing dietary components, making it challenging to maintain optimal performance across diverse and complex matrices.

Conversely, concerning inhibition-type mechanisms, particularly those utilizing biological agents, distinct challenges arise. Although these agents often exhibit superior AFs removal capabilities in the in vitro studies, their biological activity and stability are frequently challenged within complex food or feed environments. For example, *Lactobacillus brevis DN-1*, a strain isolated from moldy feed, demonstrated a remarkable AFB1 removal rate of 71.38% and effectively inhibited the growth of *A. flavus* and *Aspergillus parasiticus*. However, this study also highlighted that the strain’s optimal activity occurs at 37 °C and pH 6.0. While it retained an AFB1 removal efficiency exceeding 60% under anaerobic conditions across a range of 20–40 °C and pH 3.0–9.0 [170], significant hurdles remain for large-scale application. Maintaining optimal strain viability, ensuring long-term stability across varying matrices, and managing competition from indigenous microbial communities present substantial technical challenges. Consequently, the potency of a single biological inhibitor may be insufficient to completely mitigate AFs risks, particularly under conditions of high contamination load. These inherent limitations underscore the imperative for developing multifunctional, high-efficiency, and broad-spectrum strategies for AFs control.

Given the inherent limitations of single-mechanism approaches, the synergistic integration of diverse strategies has emerged as a critical frontier in addressing the challenge of AFs contamination. By coupling adsorption-type with inhibition-type mechanisms, complementary advantages can be realized, significantly enhancing AFs removal efficiency while simultaneously attenuating toxicity.

A prime exemplar of this synergy is the combined application of biochar and probiotics [171,172]. A recent study investigated the efficacy of Prosopis farcta biochar in mitigating the adverse effects of AFB1 in quails. In the in vitro studies confirmed the superior adsorption capacity of this biochar for AFB1. In a 35-day feeding trial, dietary supplementation with biochar alone significantly improved growth performance, immune function, and hepatic health in AFB1-exposed quails compared to controls. Crucially, the co-administration of biochar with *Lactobacillus fermentum* yielded the most promising results in alleviating the deleterious effects of AFB1 on skeletal health [173]. This finding not only validates the role of biochar as a high-efficiency adsorbent but also highlights the potential of probiotics to synergistically detoxify residues via gut microbiota modulation and immune enhancement.

Furthermore, multi-technology integration offers a broader perspective for mechanism synergy [174,175,176]. In the management of AFs contamination in pistachios, researchers have proposed comprehensive solutions that combine non-thermal processing methods, such as pulsed light and cold plasma, with nanomaterial-based adsorbents, bioactive coatings, and aptamer-based monitoring systems. Such strategies suggest that AFs control can be strengthened by integrating processing-based decontamination, adsorption, inhibition, degradation, and monitoring rather than relying on a single intervention.

Nevertheless, the prevention of fungal contamination and aflatoxin formation during field production, harvesting, post-harvest handling, processing, and storage remains the most critical strategy for protecting the safety of food and feed products. This requires the implementation of good agricultural practices (GAP), including crop rotation, the use of resistant varieties, appropriate irrigation, pest and disease control, timely harvesting, and proper drying. It also depends on good manufacturing practices (GMP), such as maintaining hygienic processing environments, controlling moisture levels, ensuring adequate ventilation, segregating high-risk batches, and applying scientifically sound storage management. By limiting fungal colonization and toxin formation before contamination becomes established, these preventive measures provide a more fundamental and sustainable approach than interventions applied only after contamination has occurred.

These prevention-oriented strategies can be further strengthened by data-driven approaches based on artificial intelligence (AI) and machine learning (ML). By integrating environmental variables, such as temperature, relative humidity, rainfall, water activity, and storage conditions, with spectral, sensor-derived, imaging, or supply-chain data [135], AI/ML models can support early prediction of Aspergillus growth and aflatoxin contamination risk [177]. Rather than functioning merely as post-contamination detection tools, these models can serve as decision-support systems for identifying high-risk fields, batches, or storage conditions, optimizing sampling frequency, and guiding timely interventions during harvesting, post-harvest handling, storage, processing, and distribution. When combined with rapid biosensing platforms or non-destructive imaging technologies, AI-assisted prediction may enable more adaptive and near-real-time monitoring of aflatoxin risks across food and feed supply chains [178].

Looking forward, the development of novel multifunctional composite materials and intelligent control systems that integrate physical adsorption, biodegradation, and enzymatic transformation represents a pivotal direction for the field of AFs control [179,180]. Such holistic systems promise to overcome current bottlenecks and provide sustainable, high-efficacy solutions for food safety.

## 5. Conclusions

Given the inherent limitations of single-mechanism strategies in addressing the multifaceted challenge of AFs contamination, this review underscores that the future of prevention and control lies in the synergistic integration of adsorption-type and inhibition-type mechanisms. This synergy effectively leverages distinct strategic advantages: high-performance adsorbents can rapidly sequester the bulk toxin load (“capture”), while biological or chemical inhibitors concurrently degrade residual toxins or blockade their toxicological pathways (“degrade”), thereby establishing a comprehensive, efficient, and safe management framework.

Future research initiatives should prioritize the development of multifunctional intelligent synergistic control systems. These systems should be capable of adaptively modulating the ratio of adsorption to inhibition strategies according to the severity and type of contamination, thereby ensuring optimal efficacy across diverse application scenarios. Furthermore, validating these synergistic strategies in complex food and feed matrices is essential. Rigorous investigations into their stability, bioavailability, and safety during processing, storage, and digestion are critical prerequisites for practical adoption. Ultimately, the synergy of these mechanisms will enable innovative and sustainable solutions that substantially reduce the global health and economic burdens posed by AFs.

## Figures and Tables

**Table 1:** Advantages and limitations of different types of adsorbents.

Adsorbent Type	Representative Materials	Advantages	Limitations	Reference
Layered clay mineral adsorbents	Montmorillonite, bentonite, clinoptilolite, and silicate-based materials	Large specific surface area; interlayer structures and surface charges favorable for AFB1 or AFM1 binding; low cost and good thermal stability, suitable for feed and certain food matrices.	Limited adsorption selectivity; possible co-binding of nutrients such as vitamins, minerals, and proteins; adsorption efficiency susceptible to pH, ionic strength, toxin concentration, and matrix composition.	[18,36,37]
Modified clay minerals	Organo-modified montmorillonite, chlorophyll-modified montmorillonite, and surfactant-modified clays	Modification can alter interlayer hydrophobicity, surface charge, and functional group distribution, thereby improving AFB1 affinity and binding stability.	Increased preparation cost; potential residues of modifying agents; need for further evaluation of safety and long-term stability.	[19,20,38]
Carbon-based or porous adsorbents	Activated carbon, biochar, and carbonized plant by-products	Rich porous structure; ability to bind certain toxins through hydrophobic interactions, and physical adsorption.	Strong non-specific adsorption; possible binding of flavor compounds, lipid-soluble nutrients, or bioactive substances; potential sensory effects in food matrices.	[39,40,41]
Inorganic–biological composite adsorbents	Bentonite + yeast cell wall, bentonite + lactic acid bacterial cell wall, and clay + β-glucan systems	Combination of high-capacity binding sites from mineral materials and multiple functional groups from biological components; good application potential under multi-mycotoxin co-contamination conditions.	Synergistic effects influenced by component ratio, particle size, surface charge, and pore structure; poorly optimized formulations may cause site shielding or enhanced non-specific adsorption of nutrients.	[37,41,42]

**Table 2:** Comparison and application scenarios of aflatoxin control mechanisms.

Parameter	Adsorption-Based Strategies	Inhibition-Based Strategies	Synergistic Approaches
Kinetics	Moderate to slow onset; sustained effect over transit time [135,136]	Rapid initial action; time-dependent bioactivity [137,138]	Sequential kinetics: immediate sequestration followed by sustained degradation [139,140]
Selectivity	Low selectivity; broad-spectrum binding capacity [141,142]	High target specificity; selective molecular recognition [143,144]	Complementary selectivity: broad capture with targeted degradation [145,146]
Matrix Compatibility	Minimal matrix disruption; potential interference with beneficial compounds [147,148]	Generally, matrix-compatible; metabolite formation requires monitoring [86,149]	Optimized to preserve matrix integrity while minimizing nutrient losses [150,151]
Operational Stability	Susceptible to desorption and release under physiological conditions [152,153]	Variable stability; influenced by environmental factors and microbial activity [57,154]	Enhanced stability through combined mechanisms; reduced post-desorption risks [73,155]
Safety Profile	Generally recognized as safe; dependent on material purity and source [156,157]	Requires comprehensive assessment of metabolites and host interactions [158]	Integrated safety evaluation addressing both components and their interactions [73]
Application Scenarios	Emergency decontamination of contaminated matrices; short-term detoxification [159]	Preventive treatment of raw materials; long-term contamination control during processing [23,154]	Comprehensive contamination management: immediate response and long-term prevention [18,160]
Primary Limitations	Non-specific binding; nutrient depletion risk; potential desorption in complex matrices [159]	Variable transformation efficiency; metabolite toxicity concerns; biosafety considerations [18,161]	System complexity; cost-benefit optimization; integration challenges [162]
Mitigation Strategies	Surface functionalization for enhanced selectivity; co-administration with nutrient supplements; process optimization [163]	Strain screening and optimization; encapsulation technologies; multi-component formulations [161]	Development of multifunctional composites; sequential dosing protocols; AI-driven monitoring systems [162]
Mechanism of Action	Physical sequestration through adsorption, interlayer trapping, electrostatic interactions, hydrogen bonding, and hydrophobic associations. Toxins are immobilized without chemical transformation.	Biological or chemical intervention targeting fungal growth inhibition, biosynthetic pathway disruption, or direct toxin degradation through enzymatic or oxidative processes.	Integrated “capture-and-degrade” systems combining rapid toxin sequestration with concurrent or sequential biological/chemical degradation.
Efficacy	Variable performance depending on matrix conditions. Representative data: Aloe powder (68.52% AFB_1_ removal), zeolite (70.19% AFB_1_ removal), sugarcane bagasse-derived aluminosilicate (88.25% AFB_1_ removal in intestinal phase) [33,164]	High efficacy in targeted applications. Examples: 2,5-dihydroxybenzaldehyde (98.7% *A. flavus* growth inhibition; AFB_1_ reduction from 994 to 1 μg/kg), piperitone (growth and biosynthesis inhibition via cellular damage and gene downregulation) [165,166]	Enhanced performance through synergistic effects. Fe-MOF–laccase–magnetic biochar system: 11-fold higher degradation capacity than free enzyme, >85% efficiency retention after five cycles; Amphipathic laccase hybrids: reduction of 50–150 μg/kg AFB_1_ to <0.96 μg/kg within 3 h [167,168]

## Data Availability

No new data were created or analyzed in this study.

## Abbreviations

The following abbreviations are used in this manuscript:

AFs	Aflatoxins
*A. flavus*	*Aspergillus flavus*
AFB1	Aflatoxin B1
AFM1	Aflatoxin M1
AF	Aflatoxin
CMCH	chlorophyll-modified calcium montmorillonite
HIGS	Host-induced gene silencing
YCW	Yeast cell wall
HA	Humic acids

## References

[1] Kumar P., Mahato D.K., Kamle M., Mohanta T.K., Kang S.G. (2017). Aflatoxins: A global concern for food safety, human health and their management. Front. Microbiol..

[2] Marin S., Ramos A.J., Cano-Sancho G., Sanchis V. (2013). Mycotoxins: Occurrence, toxicology, and exposure assessment. Food Chem. Toxicol..

[3] (2012). IARC Working Group on the Evaluation of Carcinogenic Risks to Humans. Aflatoxins. Chemical Agents and Related Occupations.

[4] Hua Z., Liu R., Chen Y., Liu G., Li C., Song Y., Cao Z., Li W., Li W., Lu C. (2021). Contamination of Aflatoxins Induces Severe Hepatotoxicity Through Multiple Mechanisms. Front. Pharmacol..

[5] Nakai V.K., de Oliveira Rocha L., Gonçalez E., Fonseca H., Ortega E.M.M., Corrêa B. (2008). Distribution of fungi and aflatoxins in a stored peanut variety. Food Chem..

[6] Zhu Z., Shahab M., Uddin S., Ishfaq M. (2025). Aflatoxin B1-Induced Male Reproductive Toxicity: Bioactivation, Mechanisms, Molecular Pathways, and Mitigation by Phytochemicals in Humans and Animals. Toxicon.

[7] Francis S., Kortei N.K., Sackey M., Richard S.A. (2024). Aflatoxin B1 induces infertility, fetal deformities, and potential therapies. Open Med..

[8] Cui R., Pan A., Wang T., Liang Y., Yu H.F. (2025). Aflatoxin B1 in animals: Metabolism and immunotoxicity. Pak. Vet. J..

[9] Alharthi S., Uguru H., Akpokodje O.I., Sami R., Alqurashi M., Aloufi S., Al-Otaibi S.A., Albaqami J.J., Zarah R.K., Hamdi H. (2025). The effect of pollution on the livestock management, microbial evaluation, health risks, and HPLC analysis of aflatoxins in animal meat and organs. Front. Sustain. Food Syst..

[10] Dyahyuningtyas H.A., Aurum F.S., Karimy M.F., Damayanti E., Rosyida V.T., Ndraha N., Fitrianto N., Siregar T.H., Dwiyitno D., Pourazad P. (2026). Untargeted Metabolomics Reveals Higher Contaminant Occurrences in Mixed-Grains of Dairy Cow Feedstuff During Wet Season of Tropical Climate Zone. Food Hum..

[11] Guo C., Zhao Y., Liu A., Wang D., Wang X., Yu L., Ma F., Wang X., Fang M., Ding X. (2026). Dynamic changes and early warning of peanuts aflatoxin B1 contamination in China in the context of climate change. npj Sci. Food.

[12] Olorunkosebi M.T., Ismaila E.O., Lagbel G., Farinde T.D., Jimoh T.S., John A.L. (2026). Climate Change and Foodborne Pathogens: A Comprehensive Review of Emerging Risks and Predictive Modelling. J. Adv. Food Sci. Technol..

[13] Liang L., Wang X., Lan H., Wei S., Lei Y., Zhang S., Zhai H., Hu Y., Lv Y. (2024). Comprehensive analysis of aflatoxin B1 biosynthesis in *Aspergillus flavus* via transcriptome-wide m6A methylome response to cycloleucine. J. Hazard. Mater..

[14] Sharma K.K., Pothana A., Prasad K., Shah D., Kaur J., Bhatnagar D., Chen Z.-Y., Raruang Y., Cary J.W., Rajasekaran K. (2018). Peanuts that keep aflatoxin at bay: A threshold that matters. Plant Biotechnol. J..

[15] Gangurde S.S., Korani W., Bajaj P., Wang H., Fountain J.C., Agarwal G., Pandey M.K., Abbas H.K., Chang P.-K., Holbrook C.C. (2024). *Aspergillus flavus* pangenome (AflaPan) uncovers novel aflatoxin and secondary metabolite associated gene clusters. BMC Plant Biol..

[16] Legan A.W., Mack B.M., Mehl H.L., Wissotski M., Ching’anda C., Maxwell L.A., Callicott K.A. (2023). Complete genome of the toxic mold *Aspergillus pseudotamarii* isolate NRRL 25517 reveals genomic instability of the aflatoxin biosynthesis cluster. G3 Genes Genomes Genet..

[17] Medalcho T.H., Abegaz K., Dessalegn E., Mate J. (2023). Aflatoxin B1 Detoxification Potentials of Garlic, Ginger, Cardamom, Black Cumin, and Sautéing in Ground Spice Mix Red Pepper Products. Toxins.

[18] Guo Y.X., Liu J.H., Gates W.P., Zhou C.H. (2020). Organo-modification of montmorillonite. Clays Clay Miner..

[19] Sun Z., Lian C., Li C., Zheng S. (2020). Investigations on organo-montmorillonites modified by binary nonionic/zwitterionic surfactant mixtures for simultaneous adsorption of aflatoxin B1 and zearalenone. J. Colloid Interface Sci..

[20] Oladele J.O., Xenophontos X., Elizondo G.M., Daasari Y., Wang M., Tamamis P., Johnson N.M., Phillips T.D. (2025). Green-engineered montmorillonite clays for the adsorption, detoxification, and mitigation of aflatoxin B1 toxicity. Toxins.

[21] Viera I., Herrera M., Roca M. (2022). Influence of food composition on chlorophyll bioaccessibility. Food Chem..

[22] Viera I., Herrera M., Roca M. (2021). In Vitro Bioaccessibility Protocol for Chlorophylls. J. Agric. Food Chem..

[23] Vazquez-Ortiz T.K., Lozano-Contreras L., Salazar A.M., Sordo M., Figueroa-Cárdenas J.d.D., Vázquez-Durán A., Méndez-Albores A. (2025). Adsorptive potential of two natural enterosorbents for removing aflatoxin B1 under simulated gastric and small intestinal conditions. Mycotoxin Res..

[24] Naqvi S.A.R., Khosa M.K.K., Zahoor A.F., Ahmad M., Abdullah A., Janjua M.R.S.A., Abidi S.A., Khalil F.M.A., Ramadan M.F., Rocha J.M. (2025). Bentonite clay in aflatoxin mitigation: Advances, efficiency and perspectives. Arch. Microbiol..

[25] Hamad G.M., Abo El-Makarem H.S., Allam M.G., El Okle O.S., El-Toukhy M.I., Mehany T., El-Halmouch Y., Abushaala M.M.F., Saad M.S., Korma S.A. (2023). Evaluation of the adsorption efficacy of bentonite on aflatoxin M1 levels in contaminated milk. Toxins.

[26] Çakir C., Turan E., Simsek A. (2023). The effects of bentonite and activated charcoal treatments on aflatoxin content (AFB1, AFB2, AFG1, and AFG2) and physicochemical characteristics of hazelnut milk. J. Food Meas. Charact..

[27] Wei Y., Sun M., Sun J., Jiang Q., Zhang B. (2024). Effect of dietary supplementation of mycotoxin adsorbent on laying performance and oviduct health of laying hens in aflatoxin B1 exposed. Agriculture.

[28] Yilmaz D.A. (2024). The content of aflatoxin M1 in the milk of cows from Turkish farms: The effect on milk quality and the effectiveness of mycotoxin binding by a clay-based adsorbent. Int. J. Vet. Med..

[29] Lashkarashvili T., Chkuaseli A. (2024). Using new adsorbent Georgian bentonite clay “Askangel” in trout feed. World J. Adv. Res. Rev..

[30] Branstad-Spates E.H., McCarthy C.S., Dooley B.C., King L.E., Bowers E.L., Tesouro A., Borrell J., Díez D., Rottinghaus G.E., Baumgard L.H. (2024). Supplementing silicoglycidol for the reduction of aflatoxin M1 in milk and biomarkers of liver dysfunction in dairy cows. JDS Commun..

[31] Zaineldin A.I., Elsebaey E., Habotta O.A., Abdo W.S., El Basuini M.F., Dawood M.A.O. (2025). Mitigating aflatoxin B1-induced growth impairment and hepatic stress in *Nile tilapia* (*Oreochromis niloticus*): Comparative efficacy of *Saccharomyces cerevisiae* and silicate-based detoxifiers. Probiotics Antimicrob. Proteins.

[32] Daković A., Marković M., Ožegović M., Rottinghaus G.E., Obradović M., Krajišnik D., Smiljanić D., Bish D.L., Krstić J. (2026). The effects of bentonite characteristics and buffer-solution composition on the adsorption of aflatoxin B1. Clays Clay Miner..

[33] Niamnuy C., Sungsinchai S., Jarernsamrit P., Devahastin S., Chareonpanich M. (2024). Synthesis and characterization of aluminosilicate and zinc silicate from sugarcane bagasse fly ash for adsorption of aflatoxin B1. Sci. Rep..

[34] Pirouz A.A., Selamat J., Sukor R., Jambari N.N. (2021). Effective detoxification of aflatoxin B1 and ochratoxin A using magnetic graphene oxide nanocomposite: Isotherm and kinetic study. Coatings.

[35] Schlösser L.M.L., Simões C.T., Sarturi J.A., Silva C.R., Laber I.F., Franco D.S.P., Mallmann C.A. (2024). Adsorption of aflatoxin B1 by different antimycotoxin additives: Bentonite, clinoptilolite, and beta-glucans extracted from yeast cell wall. Mycotoxin Res..

[36] Daković A., Matijašević S., Rottinghaus G.E., Ledoux D.R., Butkeraitis P., Sekulić Ž. (2008). Aflatoxin B1 adsorption by natural and copper modified montmorillonite. Colloids Surf. B Biointerfaces.

[37] Nešić K., Jakšić S., Popov N., Živkov-Baloš M., Pajić M., Zloh B., Polaček V. (2020). In vitro assessment of binding capacity of combined adsorbent (bentonite with yeast cell wall extracts) and aflatoxin B1. Arch. Vet. Med..

[38] Mao J., Zhou Y., Lv G., Zhou R. (2022). Simultaneous detoxification of aflatoxin B1, zearalenone and deoxynivalenol by modified montmorillonites. Molecules.

[39] Appell M., Wegener E.C., Sharma B.K., Eller F.J., Evans K.O., Compton D.L. (2023). In vitro evaluation of the adsorption efficacy of biochar materials on aflatoxin B1, ochratoxin A, and zearalenone. Animals.

[40] Hojati M., Norouzian M.A., Assadi Alamouti A., Afzalzadeh A. (2021). In vitro evaluation of binding capacity of different binders to adsorb aflatoxin. Vet. Res. Forum.

[41] Kihal A., Rodríguez-Prado M., Calsamiglia S. (2022). The efficacy of mycotoxin binders to control mycotoxins in feeds and the potential risk of interactions with nutrient: A review. J. Anim. Sci..

[42] Ghofrani Tabari D., Kermanshahi H., Golian A., Heravi R.M. (2018). In vitro binding potentials of bentonite, yeast cell wall and lactic acid bacteria for aflatoxin B1 and ochratoxin A. *Iran*. J. Toxicol..

[43] Karami-Osboo R., Shojaee AliAbadi M.H., Maham M., Shojaee AliAbadi M.A., Kerbaje B. (2026). Date palm kernel powder as a cost-effective bio-adsorbent for ochratoxin A and aflatoxins removal from wheat flour used as food. Biomass Convers. Biorefin..

[44] Batista L.H.C., Granja-Salcedo Y.T., Ferreira I.M., Souza M.G., Abreu M.J.I., Costa e Silva L.F., Koontz A., Holder V., Pettigrew J.E., Siqueira G.R. (2025). Effects of feeding mycotoxin-contaminated diets and the use of a yeast cell wall extracts mycotoxin adsorbent on ruminal and fecal microbiota of finishing beef steers. Front. Microbiol..

[45] Li X., Yang J., Huang W., Lin G., Li M., Mai K., Zhang Y. (2025). Evaluation of the combined impact of aflatoxin B1 and deoxynivalenol fed to *Penaeus vannamei* and mitigation properties provided by a yeast cell wall extract. Ecotoxicol. Environ. Saf..

[46] Yiannikouris A., Moran C., Keegan J.D., Vartiainen S., Fox U., Apajalahti J. (2025). PSVII-14 Dietary supplementation with yeast cell wall preparations reduces the deposition of aflatoxins in the liver of pigs fed diets contaminated with low levels of aflatoxin B1. J. Anim. Sci..

[47] Solovyov V.V., Marhunova A.M., Permiakova O.L., Voblikova T.V., Semenova Y.O. (2020). Yeast cell walls adsorption capacity. IOP Conf. Ser. Earth Environ. Sci..

[48] Gherbawy Y.A., AlOmari H., Al-Harthi H.F., ElDewy E., Ioan P., Elhariry H. (2025). Sustainable control of aflatoxin B1, ochratoxin A, and fumonisin B1 in poultry feed using plant extracts and clay. Sci. Rep..

[49] Nava-Ramírez M.d.J., Vázquez-Durán A., Figueroa-Cárdenas J.d.D., Hernández-Patlán D., Solís-Cruz B., Téllez-Isaías G., López-Coello C., Méndez-Albores A. (2023). Removal of aflatoxin B1 using alfalfa leaves as an adsorbent material: A comparison between two in vitro experimental models. Toxins.

[50] Oloruntola O.D., Ogunji I., Falowo A.B., Adelegan G.F., Olarotimi O.J., Oloruntola D.A., Agbede J.O. (2025). Dietary melegueta seed powder modulates growth, hepatic function, and biomarkers to counteract aflatoxin B1 toxicity in broilers. Mycotoxin Res..

[51] Tang Y., Liu X., Tang L., Dong J. (2024). Investigating the mechanism of Bacillus amyloliquefaciens YUAD7 degrading aflatoxin B1 in alfalfa silage using isotope tracing and nuclear magnetic resonance methods. Chem. Biol. Technol. Agric..

[52] Nava-Ramírez M.J., Maguey-González J.A., Gómez-Rosales S., Hernández-Ramírez J.O., Latorre J.D., Du X., López-Coello C., Hargis B.M., Téllez-Isaías G., Vázquez-Durán A. (2024). Efficacy of powdered alfalfa leaves to ameliorate the toxic effects of aflatoxin B1 in turkey poults. Mycotoxin Res..

[53] Nava-Ramírez M.d.J., Liu J., Hernández-Ramírez J.O., Hernandez-Velasco X., Latorre J.D., Vázquez-Durán A., Zhang G., Señas-Cuesta R., Gómez-Rosales S., Stein A. (2024). Exploring the effects of an alfalfa leaf-derived adsorbent on microbial community, ileal morphology, barrier function, and immunity in turkey poults during chronic aflatoxin B1 exposure. Int. J. Mol. Sci..

[54] Lee Y., Lemmetty J.M., Nihtilä H., Koivula H., Samandoulougou S., Sawadogo-Lingani H., Katina K., Maina N.H. (2024). Efficacy of aflatoxin B1 and fumonisin B1 adsorption by maize, wheat, and oat bran. Toxins.

[55] Kodape A., Kodape A., Desai R. (2025). Rice bran: Nutritional value, health benefits, and global implications for aflatoxin mitigation, cancer, diabetes, and diarrhea prevention. Food Chem..

[56] Miljanić J., Krstović S., Perović L., Kojić J., Travičić V., Bajac B. (2024). Assessment of the nutritional benefits and aflatoxin B1 adsorption properties of blackberry seed cold-pressed oil by-product. Foods.

[57] Liu Y., Xia L., Galani Yamdeu J.H., Gong Y.Y., Orfila C. (2024). Adsorption of aflatoxin B1 to corn by-products. Food Chem..

[58] Xu P., Dong S., Luo X., Wei B., Zhang C., Ji X., Zhang J., Zhu X., Meng G., Jia B. (2023). Humic acids alleviate aflatoxin B1-induced hepatic injury by reprogramming gut microbiota and absorbing toxin. Ecotoxicol. Environ. Saf..

[59] Maguey-González J.A., Nava-Ramírez M.d.J., Gómez-Rosales S., Ángeles M.d.L., Solís-Cruz B., Hernández-Patlán D., Merino-Guzmán R., Hernández-Velasco X., Figueroa-Cárdenas J.d.D., Vázquez-Durán A. (2023). Humic acids preparation, characterization, and their potential adsorption capacity for aflatoxin B1 in an in vitro poultry digestive model. Toxins.

[60] Maguey-González J.A., Nava-Ramírez M.J., Gómez-Rosales S., Ángeles M.L., Solís-Cruz B., Hernández-Patlán D., Merino-Guzmán R., Hernandez-Velasco X., Hernández-Ramírez J.O., Loeza I. (2023). Evaluation of the efficacy of humic acids to counteract the toxic effects of aflatoxin B1 in turkey poults. Front. Vet. Sci..

[61] Elsanhoty R.M., Salam S.A., Ramadan M.F., Badr F.H. (2014). Detoxification of aflatoxin M1 in yoghurt using probiotics and lactic acid bacteria. Food Control.

[62] Javan A.J., Rasouli N., Parsaeimehr M., Abdolshahi A. (2024). The efficiency of lactic acid bacteria strains isolated from cottage cheese (Khiki) to aflatoxin M1 reduction in milk. J. Microbiota.

[63] Zhu F.H., Chen X.Y., Hou L.L., Dong J.H., Liu H.W., Zhu L.Q., Chen F. (2024). Limosilactobacillus reuteri peptidoglycan alleviates aflatoxin B1-induced toxicity through adsorbing toxins and improving growth, antioxidant status, immunity and liver pathological changes in chicks. Br. Poult. Sci..

[64] Fakhrabadipour M., Khajehrahimi A.E., Haghdoost N.S., Anvar S.A., Tala M. (2023). Efficiency of Bifidobacterium bifidum and *Saccharomyces cerevisiae* for detoxification of aflatoxin M1 in skim milk. Int. J. Dairy Technol..

[65] Sanaldi K., Coban A.Y. (2023). Detoxification of aflatoxin M1 in different milk types using probiotics. An. Acad. Bras. Cienc..

[66] Das S., Chaudhari A.K., Singh V.K., Dwivedy A.K., Dubey N.K. (2023). Chitosan-based encapsulation of *Valeriana officinalis* essential oil as edible coating for inhibition of fungi and aflatoxin B1 contamination, nutritional quality improvement, and shelf life extension of *Citrus sinensis* fruits. Int. J. Biol. Macromol..

[67] Lemmetty J., Lee Y., Laitila T., Bredehorst S., Coda R., Katina K., Maina N.H. (2025). Sequestration of aflatoxin B1 by lactic acid bacteria: Role of binding and biotransformation. Food Res. Int..

[68] Xu R., Yiannikouris A., Shandilya U.K., Karrow N.A. (2023). Comparative Assessment of Different Yeast Cell Wall-Based Mycotoxin Adsorbents Using a Model- and Bioassay-Based In Vitro Approach. Toxins.

[69] Yuan D., Hong B., Zhang S., Shan S., Zhang J., Ren C. (2025). Preparation of magnetic rice husk carbon nanocomposite for efficiently extracting aflatoxin B1 from rice followed by time-resolved fluorescent immunochromatographic assay. Food Chem. X.

[70] Man Y., An J., Liu C., Sun Y., Xu X., Song C., Zhao R., He L. (2025). Effective extraction and sensitive detection of aflatoxins in wheat by polymeric ionic liquid-based magnetic adsorbent with multiple interactions. J. Chromatogr. A.

[71] Zhang C., Wang D., Wang C., Yu H., Zhong P., Dang W., Yang Y., Wang Y., Yan X. (2025). Developing a Ni-grafted magnetic nanoparticle for direct CotA capture in rapid detoxification of aflatoxin B1. J. Hazard. Mater..

[72] Zhang C., Zhou H., Cao S., Chen J., Qu C., Tang Y., Wang M., Zhu L., Liu X., Zhang J. (2024). A magnetic reduced graphene oxide nanocomposite: Synthesis, characterization, and application for high-efficiency detoxification of aflatoxin B1. Toxins.

[73] Abasi N., Faraji A.R., Davood A. (2023). Adsorptive removal of aflatoxin B1 from water and edible oil by dopamine-grafted biomass chitosan–iron–cobalt spinel oxide nanocomposite: Mechanism, kinetics, equilibrium, thermodynamics, and oil quality. RSC Adv..

[74] Ghorbani-Nejad B., Ranjbar M., Soltani M., Dini A., Karami-Mohajeri S., Lashkarizadeh M., Moradi Ghahderijani M., Mandegary A., Heidari M.R., Khazaeli P. (2025). Chitosan-functionalized bentonite nanostructure as a promising compound to reduce the toxicity of aflatoxin B1: An in vitro and in vivo study. Expert Opin. Drug Metab. Toxicol..

[75] Jabeen M., Jabeen S., Ahmed F., Asghar M.A. (2025). Development of lignin-chitosan-iron nanocomposite for effective removal of aflatoxin B1 in food and feed systems. J. Clust. Sci..

[76] Moradian M., Faraji A.R., Davood A. (2024). Removal of aflatoxin B1 from contaminated milk and water by nitrogen/carbon-enriched cobalt ferrite-chitosan nanosphere: RSM optimization, kinetic, and thermodynamic perspectives. Int. J. Biol. Macromol..

[77] Garcia Gonzalez M.N., Quiroga-Flores R., Börjesson P. (2022). Life cycle assessment of a nanomaterial-based adsorbent developed on lab scale for cadmium removal: Comparison of the impacts of production, use and recycling. Clean. Environ. Syst..

[78] Bagotia N. (2025). Regeneration strategies for exhausted adsorbents used in water treatment—A critical review. J. Water Process Eng..

[79] Barrientos Velazquez A.L., Deng Y. (2020). Reducing competition of pepsin in aflatoxin adsorption by modifying a smectite with organic nutrients. Toxins.

[80] Deng Y., Liu L., Barrientos Velázquez A.L., Dixon J.B. (2012). The determinative role of the exchange cation and layer-charge density of smectite on aflatoxin adsorption. Clays Clay Miner..

[81] Loi M., Fanelli F., Zucca P., Liuzzi V.C., Quintieri L., Cimmarusti M.T., Monaci L., Haidukowski M., Logrieco A.F., Sanjust E. (2019). Aflatoxin B1-adsorbing capability of *Pleurotus eryngii* mycelium: Efficiency and modeling of the process. Front. Microbiol..

[82] Wongtangtintan S., Saipan P., Tengjaroenkul U., Suthimun S., Tengjaroenkul B. (2014). Effect of heat treatment on efficacy of Thai bentonite for adsorption of aflatoxin B1 in vitro. Livest. Res. Rural. Dev..

[83] Barrientos-Velázquez A.L., Arteaga S., Dixon J.B., Deng Y. (2016). The effects of pH, pepsin, exchange cation, and vitamins on aflatoxin adsorption on smectite in simulated gastric fluids. Appl. Clay Sci..

[84] Alam S.S., Deng Y. (2017). Protein interference on aflatoxin B1 adsorption by smectites in corn fermentation solution. Appl. Clay Sci..

[85] Kihal A., Rodriguez-Prado M., Godoy C., Cristofol C., Calsamiglia S. (2020). In vitro assessment of the capacity of certain mycotoxin binders to adsorb some amino acids and water-soluble vitamins. J. Dairy Sci..

[86] Hernández-Martínez S.P., Delgado-Cedeño A., Ramos-Zayas Y., Franco-Molina M.A., Méndez-Zamora G., Marroquín-Cardona A.G., Kawas J.R. (2023). Aluminosilicates as a double-edged sword: Adsorption of aflatoxin B1 and sequestration of essential trace minerals in an in vitro gastrointestinal poultry model. Toxins.

[87] Li S., Qin K., Fu Y., He D., Han D., Li S., Wang Y., Ren S., Peng Y., Gao Z. (2023). Highly efficient removal of aflatoxin B1 employing a flower-like covalent organic framework-based fiber membrane. J. Environ. Chem. Eng..

[88] Kader M., Xu L., Fang L., Wufuer R., Zhang M., Wei N., Wang D., Zhang Z. (2025). The antimicrobial extract derived from Pseudomonas sp. HP-1 for inhibition of *Aspergillus flavus* growth and prolongation of maize seed storage. Foods.

[89] Yi Y., Shan Y., Lou Y., Ning Z., Zhang Q., Yang Y., Liang Y., Shi J., Hou Z. (2023). Antifungal strains M1-8 and M6-4 as biocontrol agents against *Aspergillus flavus* on peanut kernels. Qual. Assur. Saf. Crops Foods.

[90] García-Gutiérrez C., Lizárraga-Sánchez G.J., Armenta-Bojórquez A.D., Apodaca-Sánchez M.Á. (2012). Effect of biorrational products on the incidence of fungus and aflatoxins concentration in white corn grown in Sinaloa, Mexico. Rev. Cient. UDO Agríc..

[91] Shukla S., Park H.K., Lee J.S., Kim J.K., Kim M. (2014). Reduction of biogenic amines and aflatoxins in Doenjang samples fermented with various Meju as starter cultures. Food Control.

[92] Chen Y., Huang H., Li D., Zou L., Yu W., Dong W., Yu X., Feng Y., Liu J., Zhao S. (2025). Improving nutritional quality and aflatoxin detoxification of peanut meal by co-fermentation with *Weizmannia coagulans*, *Bacillus subtilis*, and supplemented enzymes. Microb. Cell Fact..

[93] Li K., Cheng S., Liu Z., Pan Q., Zuo X., Guo A., Lv J. (2024). Characteristics of inhibition of *Aspergillus flavus* growth and degradation of aflatoxin B1 by cell-free fermentation supernatant of *Bacillus velezensis* 906. Food Biosci..

[94] Purnawita W., Rahayu W.P., Lioe H.N., Nurjanah S., Wahyudi S.T. (2024). Potential molecular mechanism of reuterin on the inhibition of *Aspergillus flavus* conidial germination: An in silico study. J. Food Sci..

[95] Daba H.G., Delele M.A., Fanta S.W. (2025). Inhibition of *Aspergillus flavus* sporulation and aflatoxin production in stored maize using powdered leaf of botanicals. Arch. Phytopathol. Plant Prot..

[96] Zhang D., Luo K., Wen S., Zhou Q., Li B., Liang W., Di J. (2025). Isolation and identification of *Aspergillus* spp. from rotted walnuts and inhibition mechanism of *Aspergillus flavus* via cinnamon essential oil. Foods.

[97] Lorán S., Carramiñana J.J., Juan T., Ariño A., Herrera M. (2022). Inhibition of *Aspergillus parasiticus* growth and aflatoxins production by natural essential oils and phenolic acids. Toxins.

[98] Zhang K., Wang Q., Zhang N., Yu L., Lin Q., Zhou W. (2025). Inhibition effect of 2-ethylhexanol against *Aspergillus flavus* and aflatoxin B1 mainly by disrupting cell membrane and downregulating genes related to ergosterol synthesis and aflatoxins global regulator. Food Chem..

[99] Ling L., Mo R., Zhang W., Jiang Y., Kong F., Feng L., Li Y., Yue R., Zhou Y. (2025). Unravelling the inhibition mechanism of 1-octene-3-ol combined with 3-heptene-2-one on *Aspergillus flavus* and its application in the preservation of wolfberries. Postharvest Biol. Technol..

[100] Perry S.W., Norman J.P., Barbieri J., Brown E.B., Gelbard H.A. (2011). Mitochondrial membrane potential probes and the proton gradient: A practical usage guide. BioTechniques.

[101] Massarolo K.C., Mendoza J.R., Verma T., Kupski L., Badiale-Furlong E., Bianchini A. (2021). Fate of aflatoxins in cornmeal during single-screw extrusion: A bioaccessibility approach. LWT.

[102] Shen M.-H., Singh R.K. (2022). Decomposing aflatoxins in peanuts using advanced oxidation processes by UV and H_2_O_2_. Food Bioprocess Technol..

[103] Zhang Y., Li M., Liu Y., Guan E., Bian K. (2021). Degradation of aflatoxin B1 by water-assisted microwave irradiation: Kinetics, products, and pathways. LWT.

[104] Zhang Y., Li M., Liu Y., Guan E., Bian K. (2020). Reduction of aflatoxin B1 in corn by water-assisted microwaves treatment and its effects on corn quality. Toxins.

[105] Luo X., Wang R., Wang L., Li Y., Bian Y., Chen Z. (2014). Effect of ozone treatment on aflatoxin B1 and safety evaluation of ozonized corn. Food Control.

[106] Shi H., Cooper B., Stroshine R.L., Ileleji K.E., Keener K.M. (2017). Structures of degradation products and degradation pathways of aflatoxin B1 by high-voltage atmospheric cold plasma treatment. J. Agric. Food Chem..

[107] Zhou A., Zhang Q., Adegoke T.V., Cheng D., Neng J., Wang Y. (2025). Pulsed light inhibits aflatoxins production of *Aspergillus flavus*, degrades aflatoxin B1 and its potential mechanisms. Food Chem..

[108] Abbas A., Prajapati R.K., Aalto-Setälä E., Baykov A.A., Malinen A.M. (2024). Aflatoxin biosynthesis regulators AflR and AflS: DNA binding affinity, stoichiometry, and kinetics. Biochem. J..

[109] Liu B.H., Chu F.S. (1998). Regulation of aflR and its product, AflR, associated with aflatoxin biosynthesis. Appl. Environ. Microbiol..

[110] Wang P., Xu J., Chang P.-K., Liu Z., Kong Q. (2022). New insights of transcriptional regulator AflR in *Aspergillus flavus* physiology. Microbiol. Spectr..

[111] Chang Y., Su T., Duan M., Ding M., Wu K., Wang Z., Wu S., Duan N. (2026). Rational engineering of a polyvalent aptamer for mitigating aflatoxin B1 toxicity by disrupting metabolite aflatoxin B1-8,9-epoxide formation. J. Hazard. Mater..

[112] Xu Z., Liu Q., Liu X., Yang M., Su Y., Wang T., Li D., Li F. (2022). Integrated transcriptome analysis reveals mRNA–miRNA pathway crosstalk in Roman laying hens’ immune organs induced by AFB1. Toxins.

[113] Han Z., Migheli Q., Kong Q. (2024). Fusion expression of peptides with AflR binuclear zinc finger motif and their enhanced inhibition of *Aspergillus flavus*: A study of engineered antimicrobial peptides. J. Agric. Food Chem..

[114] Khalid M.U., Phyo H.M., Du Y., Ali K., Ashraf W., Yu H., Khan I., Yao W. (2024). Synergistic inhibition effect of anisaldehyde and cinnamaldehyde (SAC) against *Aspergillus* species (*A. flavus* and *A. niger)* in vitro and on bread. Food Biosci..

[115] Wei S., Zhang Y., Wu M., Lv Y., Zhang S., Zhai H., Hu Y. (2024). Mechanisms of methyl 2-methylbutyrate suppression on *Aspergillus flavus* growth and aflatoxin B1 biosynthesis. Int. J. Food Microbiol..

[116] Wang X., Sahibzada K.I., Du R., Lei Y., Wei S., Li N., Hu Y., Lv Y. (2024). Rhein inhibits cell development and aflatoxin biosynthesis via energy supply disruption and ROS accumulation in *Aspergillus flavus*. Toxins.

[117] Bruns H.A., Abbas H.K. (2006). Effects of glufosinate-ammonium and urea on aflatoxin and fumonisin levels in corn. Plant Health Prog..

[118] Hussein A.H., Hussein H.Z. (2024). Effect of standard and nano-urea on the inhibition of the fungus *Aspergillus flavus* growth and reduction of aflatoxin B1 production in the laboratory. Arab J. Plant Prot..

[119] Thakaew R., Chaiklangmuang S. (2023). Aflatoxin B1 elimination in low-grade maize by co-influence of heat and chemical treatment. Qual. Assur. Saf. Crops Foods.

[120] Shahadha A.F., Al-Aubadi I.M., Merzah N.R. (2024). Evaluation of the efficiency of chitosan produced from the stalks of *Agaricus bisporus* brown as an antifungal against *Aspergillus flavus* and reducing aflatoxin B1. Iraqi J. Mark. Res. Consum. Prot..

[121] Prasad J., Das S., Maurya A., Soni M., Yadav A., Singh B., Dwivedy A.K. (2023). Encapsulation of *Cymbopogon khasiana* × *Cymbopogon pendulus* essential oil (CKP-25) in chitosan nanoemulsion as a green and novel strategy for mitigation of fungal association and aflatoxin B1 contamination in food system. Foods.

[122] Aphaiso P., Mahakhan P., Sawaengkaew J. (2024). *Bacillus siamensis* 3BS12-4 extracellular compounds as a potential biological control agent against *Aspergillus flavus*. J. Microbiol. Biotechnol..

[123] Cheng X., Zhu W., Zhu X., Zhang J., Yang J., Wang H., Mo X., Zhang C., Wu L. (2025). Self-sufficient aflatoxin decontamination system: MOF-based composite membrane with peroxidase-mimic and controlled H_2_O_2_ generation. Toxins.

[124] Guan Y., Chen J., Nepovimova E., Long M., Wu W., Kuca K. (2021). Aflatoxin detoxification using microorganisms and enzymes. Toxins.

[125] Loi M., Logrieco A.F., Pusztahelyi T., Leiter É., Hornok L., Pócsi I. (2023). Advanced mycotoxin control and decontamination techniques in view of an increased aflatoxin risk in Europe due to climate change. Front. Microbiol..

[126] Kaptoge L., Ortega-Beltran A., Atehnkeng J., Konlambigue M., Kamau J.W., Bandyopadhyay R. (2024). The challenge of industrialization of a nature-based solution that allows farmers to produce aflatoxin-safe crops in various African countries. Front. Sustain. Food Syst..

[127] Kumar V., Bahuguna A., Ramalingam S., Dhakal G., Shim J.-J., Kim M. (2022). Recent technological advances in mechanism, toxicity, and food perspectives of enzyme-mediated aflatoxin degradation. Crit. Rev. Food Sci. Nutr..

[128] Jia M., Yu X., Xu K., Gu X., Harmer N.J., Zhao Y., Xiang Y., Sheng X., Li C., Du X.-D. (2024). The high-efficiency degradation of multiple mycotoxins by Lac-W laccase in the presence of mediators. Toxins.

[129] Mei X., Hou M., Muzaffar N., Logrieco A.F., Mule G., Xie Y., Yang Y., Jia H., Liang Y. (2026). Rational engineering of a bifunctional laccase with enhanced activity and safety for simultaneous detoxification of aflatoxin B1 and zearalenone. Food Biosci..

[130] Wang X., Bai Y., Huang H., Tu T., Wang Y., Wang Y., Luo H., Yao B., Su X. (2019). Degradation of aflatoxin B1 and zearalenone by bacterial and fungal laccases in presence of structurally defined chemicals and complex natural mediators. Toxins.

[131] Choi D., Alshannaq A.F., Yu J.-H. (2024). Safe and effective degradation of aflatoxins by food-grade culture broth of *Aspergillus oryzae*. PNAS Nexus.

[132] Fang Q., Du M., Chen J., Liu T., Zheng Y., Liao Z., Zhong Q., Wang L., Fang X., Wang J. (2020). Degradation and detoxification of aflatoxin B1 by tea-derived *Aspergillus niger* RAF106. Toxins.

[133] Iram W., Anjum T., Iqbal M., Ghaffar A., Abbas M. (2016). Structural elucidation and toxicity assessment of degraded products of aflatoxin B1 and B2 by aqueous extracts of *Trachyspermum ammi*. Front. Microbiol..

[134] Wu Y., Cheng J.-H., Sun D.-W. (2021). Blocking and degradation of aflatoxins by cold plasma treatments: Applications and mechanisms. Trends Food Sci. Technol..

[135] Gharibzahedi S.M.T., Savas S. (2025). Processing and real-time monitoring strategies of aflatoxin reduction in pistachios: Innovative nonthermal methods, advanced biosensing platforms, and AI-based predictive approaches. Foods.

[136] Palade L.M., Dore M.I., Marin D.E., Rotar M.C., Taranu I. (2021). Assessment of food by-products’ potential for simultaneous binding of aflatoxin B1 and zearalenone. Toxins.

[137] Yue X., Ren X., Fu J., Wei N., Altomare C., Haidukowski M., Logrieco A.F., Zhang Q., Li P. (2022). Characterization and mechanism of aflatoxin degradation by a novel strain of *Trichoderma reesei* CGMCC3.5218. Front. Microbiol..

[138] Zhang A., Yang J. (2025). A review of research progress on the microbial or enzymatic degradation and mechanism of aflatoxin B1. J. Microbiol. Biotechnol..

[139] Wu W., Lu S., Jiang S., Chen J., Zheng Z., Jiang S., Yang P. (2024). Immobilization of recombinant Trametes versicolor aflatoxin B1-degrading enzyme (TV-AFB1D) with montmorillonite for absorption and in situ degradation of aflatoxin B1. Mycotoxin Res..

[140] Yang X., Yao M., Liao W., Li X. (2025). Adsorption–degradation integrated approaches to mycotoxin removal from food matrices: A comprehensive review. Toxins.

[141] Ndiaye S., Zhang M., Fall M., Ayessou N.M., Zhang Q., Li P. (2022). Current review of mycotoxin biodegradation and bioadsorption: Microorganisms, mechanisms, and main important applications. Toxins.

[142] Pérez-Álvarez M.d.C., Arroyo-Manzanares N., Campillo N., Viñas P. (2024). Magnetic molecularly imprinted polymers for selective extraction of aflatoxins from feeds. Toxins.

[143] Aasa O.A., Govender S.E., Malgas S., Thantsha M.S. (2026). Microbial and enzymatic biodegradation of aflatoxins and ochratoxins: Mechanisms, applications, and emerging innovations. Arch. Microbiol..

[144] Söylemez T., Berger R.G., Krings U., Yamaç M. (2025). Aflatoxin B1 (AFB1) biodegradation by a lignolytic phenoloxidase of *Trametes hirsuta*. Sci. Rep..

[145] Gordi Z., Teilaghi S. (2025). Novel Ni/Fe-MIL-53@ZnO nanocomposite for efficient photodegradation of aflatoxins G1 and G2. Sci. Rep..

[146] Peng Y., Mu S., Ge J. (2026). Ligand screening for enzyme immobilization enables efficient removal of aflatoxin B1 in continuous flow system. Toxins.

[147] Gemede H.F. (2025). Toxicity, mitigation, and chemical analysis of aflatoxins and other toxic metabolites produced by *Aspergillus*: A comprehensive review. Toxins.

[148] Ma R., Peng W., Xie Y., Muzaffar N., Ma W., Yang Y., Li Q., Jia H. (2025). Mechanistic insights into aflatoxin B1 and M1 degradation by *Bacillus subtilis* HNGD-Mq02 from wheat koji and its application in food detoxification. J. Agric. Food Chem..

[149] Kibugu J., Munga L., Mburu D., Maloba F., Auma J.E., Grace D., Lindahl J.F. (2024). Dietary mycotoxins: An overview on toxicokinetics, toxicodynamics, toxicity, epidemiology, detection, and their mitigation with special emphasis on aflatoxicosis in humans and animals. Toxins.

[150] Delgado-Cedeño A., Hernández-Martínez S.P., Ramos-Zayas Y., Marroquín-Cardona A.G., Méndez-Zamora G., Franco-Molina M.A., Kawas J.R. (2022). Insoluble chitosan complex as a potential adsorbent for aflatoxin B1 in poultry feed. Front. Mater..

[151] Faraji A.R., Gil A., Farahanipour A., Tehrani E., Khoramdareh N.B., Dashtabadi E., Jafari S.Z., Shojaei N., Hekmatian Z., Saeedi S. (2025). Synergic removal of aflatoxin B1 in oily matrices by focusing on the peroxidase-like nanozymes-driven strategies: Mechanisms and intermediate toxicity, nutritional impact, advances and challenges. Trends Food Sci. Technol..

[152] Ouyang B., Xu W., Ni D., Zhang W., Ding J., Mu W. (2025). Microbial and enzymatic strategies for aflatoxin control: Integrating intelligent detection and computational design. Food Chem..

[153] Xu G., Yu A., Yin W., Sun L., Zheng Y., Xu M., Zhang S., Song Y., Pu X., Cao Y. (2026). Enzymatic degradation of aflatoxin B1 by Bacillus species: Mechanistic insights and application potential in cereal detoxifications. Food Chem..

[154] Ranftler C., Zehentner M., Tschegg C., Nagl D. (2025). High sorption efficiency of purified clinoptilolite-tuff for aflatoxins B1 and M1: A case study in plant-based beverages and milk. Int. J. Mol. Sci..

[155] Yao L., Sun C., Lin H., Li G., Lian Z., Song R., Zhuang S., Zhang D. (2023). Enhancement of AFB1 removal efficiency via adsorption/photocatalysis synergy using surface-modified electrospun PCL-g-C3N4/CQDs membranes. Biomolecules.

[156] Bian L., Zheng M., Chang T., Zhou J., Zhang C. (2022). Degradation of aflatoxin B1 by recombinant laccase extracellular produced from *Escherichia coli*. Ecotoxicol. Environ. Saf..

[157] Ning Y.-Q., Peng Z., Zhang Y., Refaie A., Ge J.-H., Guo L.-J., Yang W.-J., Sun L.-H. (2025). A novel mycotoxin-degrading enzyme complex can biodegrade AFB1, DON, and ZEN co-contamination in both in vitro and in vivo experiments. Toxicon.

[158] Atanda S.A., Agunbiade F.O., Shaibu R.O. (2026). Green chemistry approach for sustainable aflatoxin remediation: Chitosan-copper nanoparticles from agricultural waste with future AI integration potential. Discov. Chem..

[159] Adeniji O.O., Akinmoladun O.F., Njobeh P.B. (2025). Microbial interventions for aflatoxin control in food systems: A 25-year global bibliometric analysis (2000–2024) with implications for food security and smart agriculture. Food Saf. Risk.

[160] Yu M., Liu P. (2023). Discussion on emergency management of food safety from the perspective of foodborne diseases caused by mycotoxins. Food Sci. Technol..

[161] Vázquez-Durán A., Nava-Ramírez M.d.J., Téllez-Isaías G., Méndez-Albores A. (2022). Removal of aflatoxins using agro-waste-based materials and current characterization techniques used for biosorption assessment. Front. Vet. Sci..

[162] Cuccato M., Amminikutty N., Spalenza V., Conte V., Bagatella S., Greco D., D’Ascanio V., Gai F., Schiavone A., Avantaggiato G. (2025). Innovative mycotoxin detoxifying agents decrease the absorption rate of aflatoxin B1 and counteract the oxidative stress in broiler chickens exposed to low dietary levels of the mycotoxin. Toxins.

[163] Stoev S.D. (2025). Biocontrol agents and natural feed supplements as a safe and cost-effective way for preventing health ailments provoked by mycotoxins. Foods.

[164] Zavala-Franco A., Hernández-Patlán D., Solís-Cruz B., López-Arellano R., Tellez-Isaias G., Vázquez-Durán A., Méndez-Albores A. (2018). Assessing the aflatoxin B1 adsorption capacity between biosorbents using an in vitro multicompartmental model simulating the dynamic conditions in the gastrointestinal tract of poultry. Toxins.

[165] Commey L., Mechref Y., Burow M., Mendu V. (2024). Identification and characterization of peanut seed coat secondary metabolites inhibiting *Aspergillus flavus* growth and reducing aflatoxin contamination. J. Agric. Food Chem..

[166] Huang X., Wu X., Zhu Y., Wang J., Wang L., Wang S. (2024). Unraveling the antifungal and anti-aflatoxin B1 mechanisms of piperitone on *Aspergillus flavus*. Food Microbiol..

[167] Lu T., Fu C., Xiong Y., Zeng Z., Fan Y., Dai X., Huang X., Ge J., Li X. (2023). Biodegradation of aflatoxin B1 in peanut oil by an amphipathic laccase–inorganic hybrid nanoflower. J. Agric. Food Chem..

[168] Rasheed U., Ul Ain Q., Liu B. (2024). Integration of Fe-MOF-laccase-magnetic biochar: From rational designing of a biocatalyst to aflatoxin B1 decontamination of peanut oil. Chemosphere.

[169] Rozovenko M.V., Ktsoeva I.I., Gazzaeva M.S., Kubatieva Z.A., Baeva A.A. (2024). Effect of antioxidant and adsorbent on the digestibility and digestibility of nutrients in broiler diets while reducing the risk of aflatoxicosis. Genet. Breed. Anim..

[170] Wang X., Wang S., Xu J., Wu B., Hu Z., Niu H. (2024). Isolation, characterization, and biopreservation of Lactobacillus brevis DN-1 to inhibit mold and remove aflatoxin B1 in peanut and sunflower cakes. Agriculture.

[171] Rashidi N., Khatibjoo A., Taherpour K., Akbari-Gharaei M., Shirzadi H. (2020). Effects of licorice extract, probiotic, toxin binder and poultry litter biochar on performance, immune function, blood indices and liver histopathology of broilers exposed to aflatoxin-B1. Poult. Sci..

[172] Raz M., Bagherzadeh-Kasmani F., Karimi-Torshizi M.A., Ghazaghi M., Mokhtarpour A., Mehri M. (2025). Prosopis farcta biochar neutralizes aflatoxin B1 and enhances health and productivity in quails. Discov. Anim..

[173] Raz M., Bagherzadeh-Kasmani F., Karimi-Torshizi M.A., Ghazaghi M., Mokhtarpour A., Mehri M. (2025). Boosting antioxidant defense and enhancing product quality by biochar and probiotics under chronic aflatoxicosis in quails. Poult. Sci..

[174] Basso A.B.G., Ali S., Corassin C.H., Rosim R.E., Oliveira C.A.F. (2023). Individual and combined decontamination effect of fermentation and ultrasound on aflatoxin B1 in wheat-based doughs: A preliminary study. Qual. Assur. Saf. Crops Foods.

[175] Gelaye Y., Luo H. (2025). Green-synthesized nanomaterials for aflatoxin mitigation: A review. Nanotechnol. Sci. Appl..

[176] Syraji Y., Jeyaramraja P.R., Mada T., Gobikanila K. (2025). Comprehensive review of aflatoxin contamination, its occurrence, effects, management, and future perspectives. Discov. Food.

[177] Focker M., Liu C., Wang X., van der Fels-Klerx H.J. (2025). The use of artificial intelligence to improve mycotoxin management: A review. Mycotoxin Res..

[178] Branstad-Spates E.H., Castano-Duque L., Mosher G.A., Hurburgh C.R., Owens P., Winzeler E., Rajasekaran K., Bowers E.L. (2023). Gradient boosting machine learning model to predict aflatoxins in Iowa corn. Front. Microbiol..

[179] Fu C., Hou L., Chen D., Huang T., Yin S., Ding P., Liao Q., Huang X., Xiong Y., Ge J. (2024). Targeted detoxification of aflatoxin B1 in edible oil by an enzyme-metal nanoreactor. J. Agric. Food Chem..

[180] Tang F., Ma F., Wang D., Fang M., Zhang L., Li P., Yu L. (2026). Highly efficient detoxification of aflatoxin B1 in peanut meals by attapulgite-supported laccase. Food Control.

